# IRSp53 senses negative membrane curvature and phase separates along membrane tubules

**DOI:** 10.1038/ncomms9529

**Published:** 2015-10-15

**Authors:** Coline Prévost, Hongxia Zhao, John Manzi, Emmanuel Lemichez, Pekka Lappalainen, Andrew Callan-Jones, Patricia Bassereau

**Affiliations:** 1 Institut Curie, Centre de Recherche, 75248 Paris Cedex 05, France; 2 CNRS, Physico-Chimie Curie, UMR 168, 75248 Paris Cedex 05, France; 3 Université Pierre et Marie Curie, 75252 Paris Cedex 05, France; 4 Université Paris-Diderot, 75205 Paris Cedex 05, France; 5 Institute of Biotechnology, University of Helsinki, 00014 Helsinki, Finland; 6 INSERM, U1065, UNSA, Centre Méditerranéen de Médecine Moléculaire, C3M, 06204 Nice Cedex 3, France; 7 CNRS, Laboratoire Matière et Systèmes Complexes, UMR 7057, 75205 Paris Cedex 13, France

## Abstract

BAR domain proteins contribute to membrane deformation in diverse cellular processes. The inverted-BAR (I-BAR) protein IRSp53, for instance, is found on the inner leaflet of the tubular membrane of filopodia; however its role in the formation of these structures is incompletely understood. Here we develop an original assay in which proteins are encapsulated in giant unilamellar vesicles connected to membrane nanotubes. Our results demonstrate that I-BAR dimers sense negative membrane curvature. Experiment and theory reveal that the I-BAR displays a non-monotonic sorting with curvature, and expands the tube at high imposed tension while constricting it at low tension. Strikingly, at low protein density and tension, protein-rich domains appear along the tube. This peculiar behaviour is due to the shallow intrinsic curvature of I-BAR dimers. It allows constriction of weakly curved membranes coupled to local protein enrichment at biologically relevant conditions. This might explain how IRSp53 contributes *in vivo* to the initiation of filopodia.

## Abstract (web summary)

The inverted-BAR domain protein IRSp53 associates with the inner leaflet of tubular membranes such as filopodia. Here, Prévost *et al*. demonstrate that the I-BAR domain of IRSp53 senses negative membrane curvature, and undergoes phase separation which may aid its clustering upon filopodia generation.

## Graphical abstract

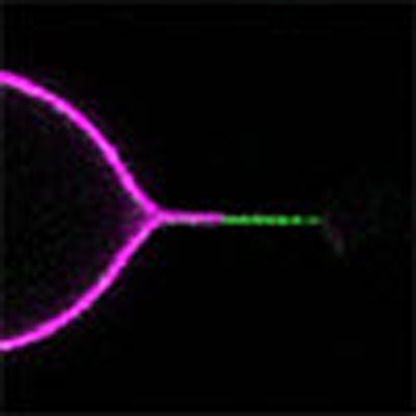

BAR (Bin/amphiphysin/Rvs) domain proteins are linked to essential cellular processes involving membrane remodelling, such as cell migration and membrane trafficking^1^. Trafficking events require the generation of highly curved membrane carriers, that is, small vesicles or narrow tubules protruding into the cytosol. BAR (including N-BARs with N-terminal amphipathic helices) and F-BAR domains generally form homodimers with an intrinsically curved concave membrane-binding interface with which they contribute to the formation of trafficking vesicles or tubules^2^,^3^. They are recruited to the neck of budding vesicles and control the later recruitment of proteins such as dynamin^4^; they also assemble into scaffolds to deform membranes, for instance in yeast^5^. Their sensor/bending behaviour depends on their density on the membrane^6^, which varies according to cell type.

Unique among the members of the BAR domain superfamily, inverted-BAR (I-BAR) domain dimers possess a convex membrane-binding interface. They have a strong structural similarity to BAR and F-BAR domains^7^, being elongated dimers with each monomer made up of a three-helix bundle, although their overall shape is markedly flatter than BARs (refs 2, 3). Consistent with the convex geometry of their membrane-binding interface, they generate membrane invaginations when bound to the outer leaflet of artificial liposomes^8^,^9^,^10^. In contrast, BAR and F-BAR generate membrane protrusions in similar assays^11^. However, the curvature generated by I-BAR and F-BAR domain proteins is shallow in general (tubule diameter 40–60 nm)^8^,^12^, whereas BAR and N-BAR domains produce much more pronounced bending (tubule diameter ∼20 nm)^13^,^14^. It is not clear yet if this structural difference may lead to functional differences.

IRSp53 (Insulin Receptor tyrosine kinase Substrate Protein of 53 kDa) is the best-studied member of the I-BAR family. It has a modular structure, comprising the N-terminal I-BAR domain, a partial CRIB motif (Cdc42/Rac-interactive binding motif), which binds the small GTPase Cdc42, and an SH3 domain, which recruits a number of regulators of actin polymerization (such as vasodilator-stimulated phosphoprotein (VASP) (ref. 15), Mena (ref. 16), Eps8 (ref. 17), mDia1 and WASP-family verprolin-homologous protein 2 (WAVE2) (ref. 18))^19^,^20^. IRSp53 also binds to the plasma membrane through its I-BAR domain, and thus constitutes a functional platform at the interface between the plasma membrane and the actin cytoskeleton^21^. Together with its binding partners^15^,^16^,^17^,^18^, it is involved in the Cdc42-dependent formation of filopodia, which are finger-like membrane protrusions containing actin bundles^22^ with a diameter typically ranging between 100 nm and a few hundred nm (ref. 23). Filopodia formation is impaired on IRSp53 inhibition^15^. Overexpression of the I-BAR domain results in the formation of plasma membrane protrusions^8^,^10^,^24^, which are uncoupled from bundled actin filaments^21^,^25^. This suggests that at high-density I-BAR domains can scaffold cell membranes. When the full-length protein is expressed at endogenous level, IRSp53 clusters have been observed at the plasma membrane preceding further recruitment of actin regulators and filopodia extension^15^. Nevertheless, the mechanism behind filopodia initiation involving IRSp53 spatial localization remains obscure. For example, whether I-BAR domains can sense negative membrane curvature through their convex geometry has not been examined. In the case of N-BAR domain proteins, systematic biophysical studies have characterized the reciprocal relation between membrane curvature and protein density^26^,^27^. A similar approach would help in deciphering the *in vivo* function of the I-BAR domain of IRSp53, but does not yet exist^15^.

To address how IRSp53 couples to membrane curvature, we used an *in vitro* system that has largely been exploited to study the effect of curvature on lipid^28^,^29^ and protein sorting^26^,^27^,^30^,^31^. By pulling membrane tubes of controlled radius from giant unilamellar vesicles (GUV; Fig. 1), and using confocal fluorescence microscopy, we are able to measure curvature-induced sorting of the I-BAR domain. This set-up further enables us to record changes in the mechanical properties of the system that occur on binding of the protein to the membrane. To mimic the cellular localization of the protein with respect to curved membranes (facing the interior of membrane tubules), a new method of encapsulation was developed, which does not require the use of hydrophobic solvents that contaminate the membrane bilayer^32^.

In this study, we combine experiments with a general theoretical description to investigate the coupling between negative membrane curvature and I-BAR domain density. We find that protein sorting depends non-monotonically on tube curvature, with an optimum value at an intrinsic curvature associated with the protein, 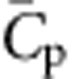. Our experiments and model also point to the capacity of these proteins to scaffold membranes at moderate density on the flat membrane. Strikingly, we discover that protein-decorated tubes may undergo phase separation into coexisting domains of different curvatures. Our work shows that this results from a mechanical coupling between the shallow I-BAR and the curved tube, independently of direct interactions between proteins. This phase separation might have implications for the role of IRSp53 in the generation of filopodia.

## Results

### IRSp53 I-BAR is enriched on negatively curved membranes

It was previously shown^8^ that the I-BAR domain of IRSp53 efficiently binds liposomes containing the negatively charged lipid L-α phosphatidylinositol-4,5-bisphosphate (PI(4,5)P_2_), the most abundant phosphoinositol in the cytosolic leaflet of the plasma membrane^33^. Here we use GUVs containing 8% PI(4,5)P_2_ (mol mol^−1^), supplemented with 10% 1,2-dioleoyl-sn-glycero-3-phospho-L-serine (DOPS). This lipid composition guarantees binding of the I-BAR domain to the membrane, and was used throughout the study. A small fraction of a lipidated dye (BodipyTR Cer, red-emitting) was incorporated in the lipid mixture, enabling to detect the membrane with confocal fluorescence microscopy. The I-BAR domain was labelled with Alexa 488 (green-emitting dye) and was detected in a separate channel.

To measure potential protein enrichment due to membrane curvature, we pulled membrane nanotubes from GUVs. Briefly, a single GUV is aspirated in a micropipette, with the adjustable pressure difference between the interior of the pipette and the experiment chamber setting the membrane tension of the GUV. A micron-sized bead trapped in an optical tweezers is then used to mechanically pull the membrane nanotube^26^,^31^. The radius of the nanotube *R* is directly set by the membrane tension 
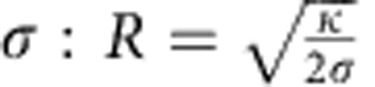
 with *κ* the bending rigidity of the membrane. *R* typically ranges from ∼7 to 100 nm in the case of bare nanotubes (that is, in the absence of proteins bound to the tubes). Our system thus consists of a large membrane reservoir with a quasi-zero curvature connected to a highly curved membrane tube, making it ideal to study curvature-induced protein sorting^6^.

**Figure 1:**
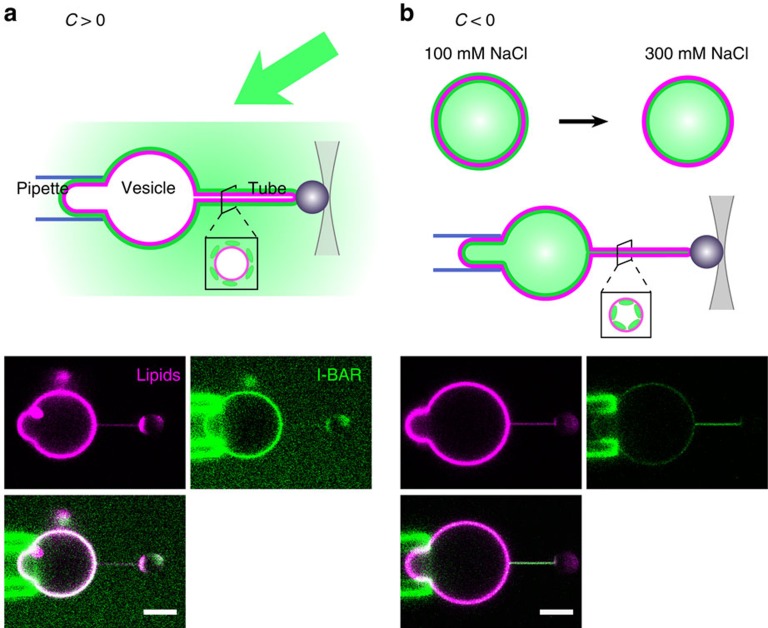
IRSp53 I-BAR dimers have a strong preference for negative membrane curvature. (**a**) Interaction of the I-BAR with positive membrane curvature. Top: A single GUV is aspirated in a micropipette (blue lines), which allows controlling its membrane tension. A bead (grey sphere) trapped in an optical tweezers (grey beam) is used to pull a membrane nanotube from the GUV. The protein is injected outside the GUV (green arrow), and therefore interacts with the positively curved outer leaflet of the tube (box). Bottom: representative confocal microscopy image. (Note that green fluorescence on the pipette results from adhesion of free protein to the glass.) Scale bar, 5 μm. (**b**) Interaction of the I-BAR with negative membrane curvature. Top: GUVs are grown in the presence of IRSp53 I-BAR dimers in 100 mM NaCl. The protein binds to both the inner and outer leaflets of GUVs. The vesicles are then transferred into a 300 mM buffer, driving the detachment of most of the proteins bound to the outer leaflet. A tube is then pulled from the GUV as in **a**. In this assay, the protein interacts with the negatively curved inner leaflet of the nanotube (box). Bottom: representative confocal image. Scale bar, 5 μm.

From its structure and its ability to induce membrane deformations with a negative curvature^8^, we hypothesized that IRSp53 may have a high affinity for negatively curved membranes, and conversely a very low affinity for positively curved membranes. Here the curvature of the membrane *C* is defined as being positive if the membrane is locally bent towards the protein solution, and negative if it is bent away. For a tube, membrane curvature is thus positive for a protein binding to the external leaflet, and negative for a protein binding to the internal leaflet (see sketches of the tube cross-section at the top of Fig. 1a,b, respectively). Following an established protocol^26^,^34^, we first checked the absence of protein enrichment on positively curved membranes: we pulled membrane tubes from GUVs, and subsequently micro-perfused the protein near the GUVs (Fig. 1a, top). We found that the fluorescence signal of the protein on the tube was very weak, suggesting that the I-BAR domain indeed has a low affinity for positive curvature (Fig. 1a, bottom).

With the tube-pulling experiment, it is in principle possible to study the affinity of proteins for negative curvature, provided that these proteins are present inside the GUV. Different methods have been reported for protein encapsulation in GUVs, either using transfer of inverted emulsion droplets covered with a lipid monolayer through a second monolayer at an oil–water interface^35^,^36^,^37^,^38^, jetting of a protein solution through a black lipid film^39^, or spontaneous GUV swelling on a polymer gel^40^,^41^. Although the first method appears promising, oil residues are trapped in the bilayer during the passage through the interface, compromising the mechanical properties of the membrane^32^. The two other methods are not suitable as well, because of oil present in black films and the large fraction of multilamellar vesicles, respectively. We thus designed a new assay to encapsulate the I-BAR domain inside unilamellar giant liposomes with controlled mechanical properties. We used an electroformation technique that was developed to enable the production of GUVs in buffers containing physiologically relevant salt concentrations^42^, in contrast with the original method^43^. We thus grew GUVs in the presence of the protein in an appropriate buffer containing 100 mM NaCl. After this stage, the protein is present both inside and outside the GUVs, and can thus bind symmetrically to both leaflets (Fig. 1b, top).

However, since the I-BAR binds to lipid bilayers mostly through electrostatic interactions^9^, screening these interactions should drive net desorption of the protein. Indeed, we verified that GUVs decorated with the I-BAR on their outer leaflet only, displayed almost complete detachment of the protein on transfer to a high-salt (300 mM NaCl) buffer solution. We estimate the residual protein area fraction to be around ∼0.5% (Supplementary Fig. 1). Thus, for our tube-pulling experiments, we transfer the freshly prepared GUVs into an external buffer containing 300 mM NaCl, to only retain vesicles containing I-BAR bound to the inner leaflet (Fig. 1b, top). With this method, a good yield of GUVs can easily be obtained, with I-BAR domains asymmetrically bound on the internal leaflet of the GUVs. In our conditions, starting from a bulk concentration of 50–100 nM, we obtained a wide range of protein density on the membrane (from about 100 up to ∼1,200 proteins per μm^2^, equivalent to an area fraction of ∼1–6%, as can be estimated from the fluorescence of the protein on the GUVs (see Methods section)). This spread might be explained by a non-uniform distribution of PI(4,5)P_2_ and/or DOPS across the GUV sample obtained by electroformation^44^,^45^. Nevertheless, we bypass this issue since the protein density is measured individually on each vesicle.

We performed tube-pulling experiments on GUVs with encapsulated IRSp53 I-BAR. As evidenced by fluorescence images (Fig. 1b, bottom), the I-BAR domain is greatly enriched in the tube, demonstrating that it indeed is a sensor of negative membrane curvature. A systematic quantitative assessment of sorting versus curvature is presented hereafter.

### Optimal negative curvature for IRSp53 I-BAR sorting

The enrichment of the protein on the tube (relative to its density on the GUV), called here the sorting ratio, is expected to depend on the curvature of the tube. We next systematically investigated this dependence for varying values of the protein area fraction on the GUV, *φ*_v_. Note that, because of the residual proteins bound to the external leaflet, we slightly over-estimate *φ*_v_ in the following.

By definition, the sorting ratio is given by 
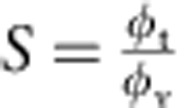
, with *φ*_t_ being the protein area fraction on the tube^26^,^31^. Both the sorting ratio and the curvature of the tube can be calculated based on fluorescence measurements: *S* corresponds to the ratio of the fluorescence intensity of the protein on the tube 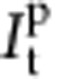 and on the GUV 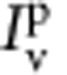, normalized by the same ratio for the lipid fluorescence: 
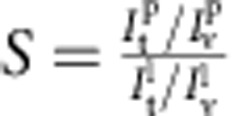
; the radius of the tube *R* (the inverse of the membrane curvature *C*) is proportional to the ratio of the fluorescence of the lipids in the tube and in the GUV^26^ (see Methods section for more details).

In a typical experiment, fluorescence and force data were collected on a stepwise increase of membrane tension (which corresponds to a stepwise decrease of tube radius). Usually these steps were then reversed, to ensure that our measurements reflected thermodynamic equilibrium (which should result in overlapping curves). We present here the data obtained in the first part of each experiment. The data obtained in the second part are shown in Supplementary Fig. 2.

**Figure 2:**
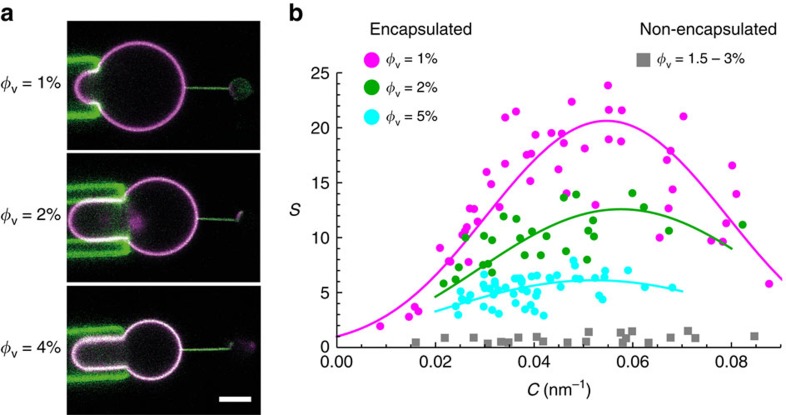
Quantification of I-BAR enrichment on highly curved membranes. (**a**) Representative confocal images at similar tensions. Top: *σ*_v_=0.11 mN m^−1^. The tube curvature is *C*=0.04 nm^−1^. Middle: *σ*_v_=0.27 mN m^−1^ (*C*=0.05 nm^−1^). Bottom: *σ*_v_=0.09 mN m^−1^ (*C*=0.03 nm^−1^) . (**b**) Sorting ratio, *S*, as a function of tube curvature, *C*=1/*R*, in the case where the protein interacts with positive (squares) and negative (circles) membrane curvature. For negative curvature, three conditions of protein area fraction *φ*_v_ on the GUVs were investigated (see Methods section): *φ*_v_≈1% (magenta, *N*=9 GUVs), *φ*_v_≈2% (green, *N*=5 GUVs) and *φ*_v_≈5% (cyan, *N*=10 GUVs). A fit to the three data sets using equation (2) yields an average protein intrinsic curvature, 
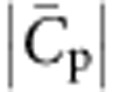
=(0.055±0.003) nm^−1^, and an average protein-membrane elastic parameter, 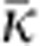
=(35±7) *k*_B_*T*. For positive curvature (grey), the protein area fraction on the GUVs was in the range 1.5–3% (*N*=4 GUVs). Scale bar, 5 μm.

Each experiment yields, for a fixed value of *φ*_v_, the values of the sorting ratio corresponding to different curvatures. Previous measurements of curvature-induced sorting of different proteins^26^,^27^,^31^,^46^ have shown that the sorting ratio at a given curvature generally depends on *φ*_v_. We therefore binned our fluorescence measurements into three ranges of *φ*_v_ (more details can be found in Methods section). Fig. 2a shows representative images for GUVs with *φ*_v_ in each range. Interestingly we found that the sorting curves display a non-monotonic shape. This feature is most striking at the lowest densities (*φ*_v_∼1%), with a maximum of sorting for *C*≈0.055 nm^−1^, that is, *R*≈18 nm (Fig. 2b, magenta points). Thus, at very low density, the I-BAR domain of IRSp53 is highly curvature-selective. The sorting effect is quite strong, since the I-BAR domain is enriched in the tube by a factor of up to ∼20. Interestingly, the curvature-selectivity is less pronounced when the protein is bound at a higher density on the GUV membrane: the relative enrichment becomes weaker and less peaked for *φ*_v_∼2 and 5% (Fig. 2b, green and cyan points, respectively), although sorting remains significant, of the order of 4–5 at *φ*_v_∼5%. We note, however, that the absolute protein density on the tube, *φ*_t_, does increase with increasing *φ*_v_; see Supplementary Fig. 3.

For comparison, we show on the same graph our data of sorting on tubes with positive curvature. The sorting ratio is between 0 and 1 for the range of curvature investigated, implying that the I-BAR domain does not have any preference for positive membrane curvature (Fig. 2b, grey points).

The dependence of IRSp53 sorting on tube curvature can be understood by thermodynamic arguments, accounting for the membrane bending and stretching energies, the protein mixing entropy, and the energetic coupling between protein and membrane^47^,^48^. Putting these elements together, the free energy of the tube decorated with proteins with area fraction *φ*_t_ is given by:


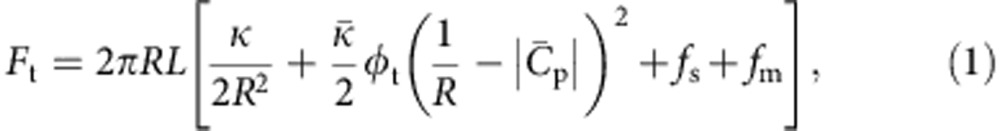


where *L* is the tube length, 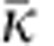 is an elastic coefficient penalizing mismatch between protein and membrane curvatures and 
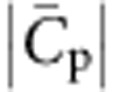
 is the membrane-bound protein's intrinsic curvature. Also, *f*_s_ and *f*_m_ are the membrane stretching and mixing energy densities, and depend on the protein and lipid densities; a particular model choice for these densities is specified in Supplementary Note 1. We note that the above protein-membrane coupling term is generic and based on symmetry, and a similar term has been used in other contexts^49^,^50^. In the absence of any other free energy penalties, the energy of a protein-membrane patch is minimum if the protein and membrane curvatures, 1/*R* and 
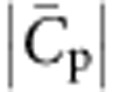
, match; to lowest order, the energy is quadratic in their difference. The mixing energy refers to the entropy of mixing of a model two-component membrane consisting of I-BAR proteins and lipids. By requiring that the lipid and protein chemical potentials on the vesicle and on the tube are equal at equilibrium (Supplementary Note 1), we obtain an implicit dependence of *φ*_t_ on the curvature:


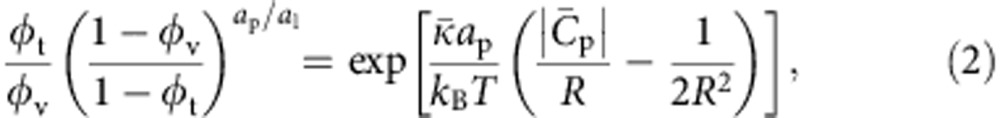


where *a*_p_ and *a*_l_ are the protein and lipid areas, respectively. At low densities, *φ*_v_ and 
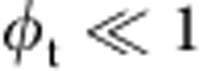
, the quantity in brackets on the left side of equation (2) tends to one, and thus the sorting *S*=*φ*_t_/*φ*_v_ has a Gaussian dependence on tube curvature *C*=−1/*R*, with a maximum at 
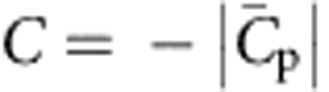
. We note that equation (2) predicts that *S* decreases with increasing *φ*_v_, in agreement with the above experimental data (Fig. 2b). Indeed, when *φ*_v_ increases, keeping all other parameters equal, the tube area fraction *φ*_t_ ultimately saturates, and thus grows more slowly than linearly with *φ*_v_.

The sorting data for IRSp53 are fitted using equation (2), with 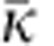 and 
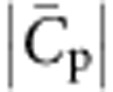
 as free parameters. The ratio *γ*=*a*_p_/*a*_l_ is, in principle, a free parameter, though does not have a strong effect on the sorting or on the mechanical effects of proteins on the tube for 
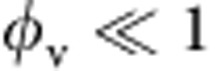
 (*γ* appears in logarithmic terms in mixing energy); see Supplementary Note 1. For simplicity of analysis, we assume throughout *γ*=1. We therefore fit the sorting data in the three different vesicle density regimes, *φ*_v_=1, 2 and 5%, (Fig. 2b), obtaining for the intrinsic curvature, 
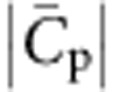
=(0.055±0.003) nm^−1^ (
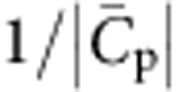
≈18 nm, averaged over the three density ranges. In addition, we find 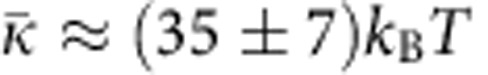. We note finally that the low value that we find for 
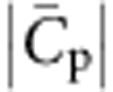
, as compared with the N-BAR protein, Amphiphysin 1 (ref. 26), is fully consistent with the known, shallow shape of IRSp53 (ref. 7).

### IRSp53 I-BAR reduces the force needed to sustain the tube

It has been shown that IRSp53 I-BAR, on binding to the outside of lipid vesicles, spontaneously forms invaginated tubes^8^. Accordingly, we found that membrane tubes spontaneously evaginate from GUVs encapsulating the I-BAR, when it is bound at sufficiently high protein coverage (see one example in Supplementary Fig. 4). Thus we expect that bound protein reduces the force, *f*, needed to hold a pre-formed membrane tube, which is a measurable parameter in our system. It can indeed be deduced from the displacement of the bead within the trap (see Methods section for more details). For bare membranes, *f* is related to the membrane-bending rigidity *κ* and the pipette-controlled tension *σ*_v_ (ref. 51) via 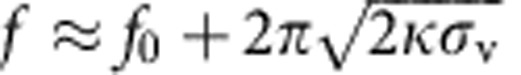, where *f*_0_ is a force offset (it takes into account effects, such as the imperfect passivation of the pipette, which could result in some degree of adhesion of the membrane to the glass, affecting membrane tension). Performing tube-pulling experiments on protein-free GUVs as a control, we obtained *κ*≈12.5 *k*_B_*T*, and *f*_0_≈5 pN (Supplementary Fig. 5a).

**Figure 3:**
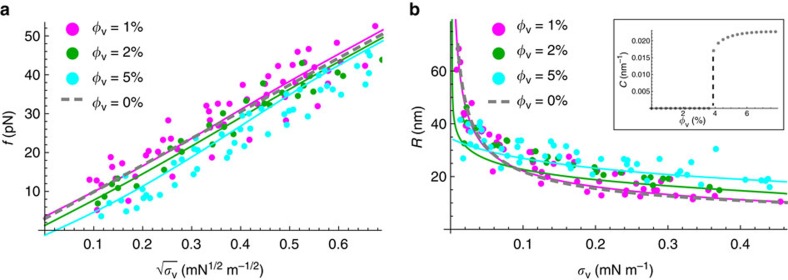
Mechanical effect of I-BAR binding to the inner leaflet of membrane tubes. (**a**) Pulling force, *f*, as a function of the square root of tension, 
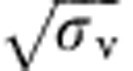
 , for three different values of *φ*_v_ : 1% (magenta, *N*=9 GUVs), 2% (green, *N*=5 GUVs) and 5% (cyan, *N*=10 GUVs). Fitting yields (see Supplementary Note 1 for details) an average value of the protein-membrane elastic parameter 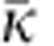
=(32±12) *k*_B_*T*. The dashed line corresponds to a fit to the data obtained for bare GUVs (Supplementary Fig. 5a). (**b**) Tube radius, *R*, as a function of *σ*_v_, for the same conditions as in (**a**). Fitting yields 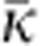
=(60±30) *k*_B_*T*. The dashed line corresponds to a fit to the data obtained for bare GUVs (Supplementary Fig. 5b). Data and fit for *φ*_v_=5% indicate that *R* extrapolates to a finite value at zero tension, whereas for *φ*_v_=1 and 2% it diverges. Inset shows calculated jump in tube curvature (1/*R*) at zero tension as a function of *φ*_v_.

We then measured the pulling force in the presence of I-BAR. We observed that, for given *σ*_v_, *f* steadily decreases with respect to the bare membrane reference as the protein density *φ*_v_ increases; see Fig. 3a. These measurements indicate that, with increasing *φ*_v_, proteins have a greater mechanical effect on the curved membrane and, in fact, stabilize the tube. This effect is seen very clearly at *φ*_v_∼5% and at low membrane tension: in these conditions, the tube becomes floppy, indicating that the pulling force is zero (see Supplementary Fig. 2b, lower inset).

In the presence of proteins, the tube force, as a function of applied tension, is calculated by minimizing the free energy, equation (1), with respect to tube length, *L*, at fixed radius and at fixed lipid and protein number; see Supplementary Note 1. In this calculation, *φ*_t_ is given by its equilibrium expression and the tube radius by the value that satisfies radial force balance on a tube element; see below and Supplementary Note 1. As a result, the dependence of *f* on *σ*_v_ is non-trivial, and must in general be found numerically. In this way, the force data are fitted for the three values of *φ*_v_, yielding 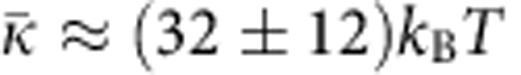; see Fig. 3a. As expected, for a given *σ* the force shifts downwards as *φ*_v_ increases, indicating a stabilizing influence of the proteins.

We can understand the dependence of *f* on *φ*_v_ more naturally if we assume that 
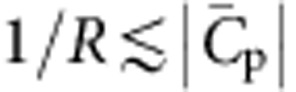
, *φ*_v_ is not negligible but still much <1, and 
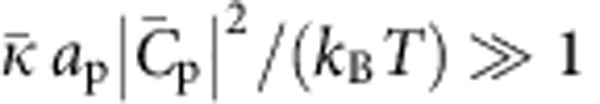
 (so that the tube is nearly saturated with proteins). We then obtain


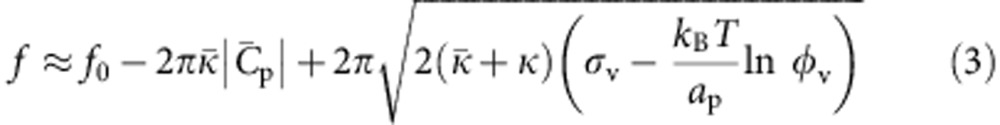


where *a*_p_≈50 nm^2^ is the area per protein^7^.

From this equation we note that *f* decreases with increasing *φ*_v_, in agreement with our data. In addition, taking the values 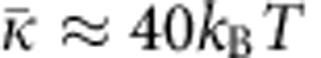 and 
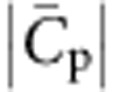
≈0.05 nm^−1^ obtained from the sorting fits, we find the offset term 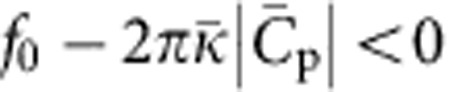 As a result, from equation (3) it is clearly seen that the force vanishes at a non-zero tension, that varies as ln *φ*_v_. Thus, our model predicts that bound IRSp53 can stabilize membrane tubes, without an external pulling force. It is, furthermore, consistent with observations of spontaneous tubulation of membranes by IRSp53.

### Tube curvature is set by IRSp53 I-BAR at high density

The dependence of the tube radius on the protein area fraction on the GUV provides a second way to assess the mechanical effects that proteins have on pre-formed, highly curved membrane structures. In the absence of protein, *R* has a straightforward relationship to GUV membrane tension *σ*_v_: 
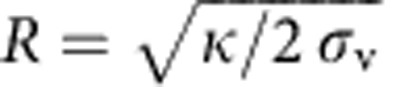
. This expression is used to fit the data obtained with bare GUVs (Supplementary Fig. 5b). In the presence of protein, we found that the radius, as a function of applied tension, clearly deviates from the bare membrane reference as the protein density increases: for *φ*_v_∼1%, the curve overlaps with the data for bare tubes, indicating negligible mechanical changes to the tube. In contrast, for *φ*_v_ ∼2 and 5%, there is a marked difference between the radii of bare and protein-decorated tubes; see Fig. 3b: (a) the overall dependence of the tube radius on tension is much less pronounced, (b) the coated-tube is wider for most of the range of membrane tension explored and (c) at vanishing tension *σ*_v_→0, the tube curvature tends to a non-zero value.

At mechanical equilibrium the tube radius is found by minimizing the tube free energy, equation (1), with respect to *R* for fixed *L*, and lipid and protein number. The result of the minimization yields an equation that depends on the applied tension, *σ*_v_, and the protein area fraction on the tube, *φ*_t_, which in turns depends on *R.* As a result, for a given *σ*_v_, *R* must generally be found numerically; see Supplementary Note 1 for details. The experimental values of *R* versus *σ*_v_ for given *φ*_v_ can be fitted with the above-described theory with 
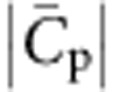
 and 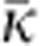 as unknown parameters in principle (
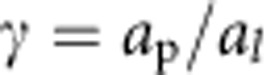
 is fixed equal to 1 as mentioned before). Since the value of 
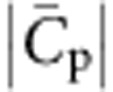
 obtained from the sorting fits across the three density regimes was constant to within <10%, we subsequently assumed 
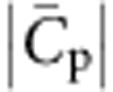
=0.052 nm^−1^. Fitting the data, we obtain 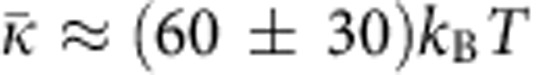.

From the tube radius fits we find that the model captures well the observed mechanical effects of bound IRSp53. First, the fits confirm that at moderately high protein densities (*φ*_v_∼5%) and at large tension (
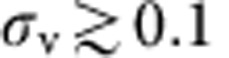
 mN m^−1^), tubes are wider than they are for bare membranes; see Fig. 3b. This feature is, on first inspection, surprising given that proteins stabilize membrane tubes, and one would expect they favour higher curvatures than that of a bare case. This behaviour can, however, be understood from an approximate expression for *R*, valid under the same assumptions leading to equation (3):


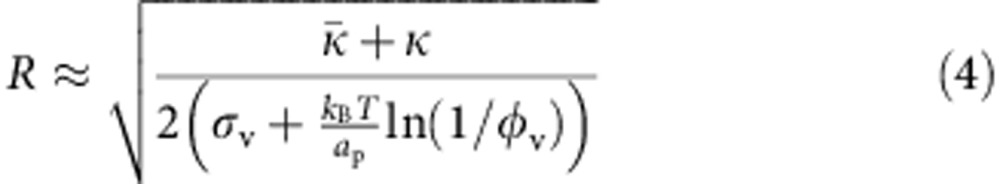


Equation (4) resembles the expression for the bare tube radius, 
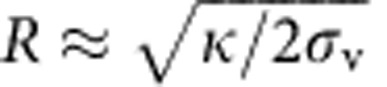
, but shows that the protein-decorated tube radius increases with increasing *φ*_v_, and that the effective rigidity is higher, given by *κ*+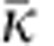. We note that in this regime, *R* is neither strongly dependent on applied membrane tension, nor is it fixed by the intrinsic curvature 
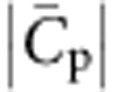
. In fact, for *φ*_v_=5% and *a*_p_≈50 nm^2^, the entropic part of the tube tension is 
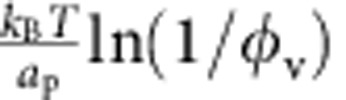
≈0.25 mN m^−1^, and therefore for most of the tensions explored 
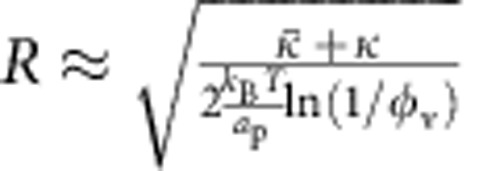
≈20 nm, assuming *κ*=15*k*_B_*T* and 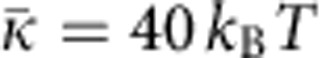, in agreement with Fig. 3b.

Second, our model confirms that at vanishing applied tension and the highest protein density (*φ*_v_≈5%) the tube curvature tends to a finite value, whereas at low densities it tends to zero, just as for a protein-free tube (Fig. 3b). By calculating the tube curvature at zero tension as a function of *φ*_v_, we see that there is a discontinuous change in the curvature at a given 
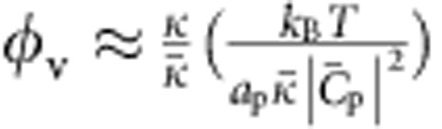
; see inset of Fig. 3b and Supplementary Note 1. This provides further quantitative proof that IRSp53 can stabilize tubes, depending on their intrinsic curvature, and consistent with their ability to tubulate flat membranes. Indeed, if a sufficient quantity of protein is bound to a floppy vesicle, highly curved structures are energetically favoured over nearly flat membrane. Finally, the discontinuous nature of curvature at vanishing tension is indicative of bistability, pointing to the possibility of coexisting phases on the tube at low tension.

### IRSp53 I-BAR phase separates on the tube

**Figure 4:**
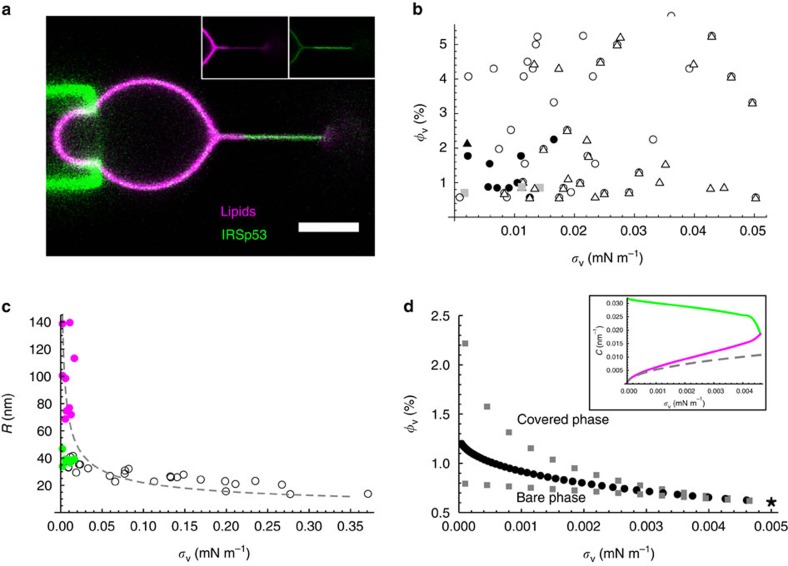
Phase separation along the tube at low membrane tension. (**a**) Confocal image showing the coexistence of two phase of the tube: a wide, protein-poor phase (magenta) and a narrow, protein-covered one (green). The inset shows the separate channels. Here *φ*_v_ ∼2% and *σ*_v_≈0.017 mN m^−1^. Scale bar, 5 μm. (**b**) Phase diagram in the *φ*_v_−*σ*_v_ plane. Filled circles (triangle) indicate phase-separated tubes observed while decreasing (increasing) tension. Open circles (triangles) indicate single-phase tubes observed while decreasing (increasing) tension. Grey squares indicate incompletely phase-separated tubes observed while decreasing tension. *N*=32 GUVs. (**c**) Tube radius, *R*, versus tension, *σ*_v_. Open circles indicate single-phase tubes (for *φ*_v_ ∼2%, *N*=5 GUVs). Magenta and green filled circles indicate radii of protein-poor and protein-covered coexisting phases, *N*=8 GUVs. The fit to the data for bare GUVs, indicated by a dashed line, is provided as a reference. (**d**) Theoretical *φ*_v_−*σ*_v_ phase diagram. Points at which nearly bare and protein-covered phases coexist are indicated by filled circles. The limits of metastability are indicated by grey squares (upper set: limit of bare phase; lower set: limit of covered phase). The predicted critical point is indicated by an asterisk. Inset shows the curvatures of phases at coexistence (same colour code as in **c**); dashed line is analytical approximation to bare phase curvature, as obtained from Supplementary Equation 33. The model parameter values are 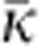
=24 *k*_B_*T*, 
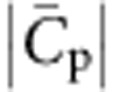
=0.055 nm^−1^, 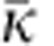
=70 *k*_B_*T*, *a*_p_=*a*_l_=50 nm^2^ and *χ*=0 (direct protein–protein interaction parameter; see Supplementary Equation 5).

We have shown so far that encapsulated IRSp53 is enriched on membrane tubes with a uniform coating of the tubes (Fig. 1b). Interestingly, at low applied tensions (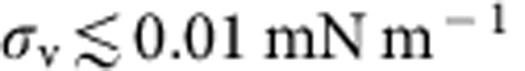) and at vesicle protein densities between 1 and 2%, we observed coexistence of two phases along the tube, with different protein densities and different radii (Fig. 4a). The protein-rich part of the tube (green in Fig. 4a) was found to have a much smaller radius than the nearly bare part (magenta).

We consistently observed protein phase separation along the tube for low tensions and low protein densities, as shown in Fig. 4b (filled circles). In cases where it was observed, phase separation was detected at the first image acquisition, typically about 1 min after a change in membrane tension; no further evolution was detected on subsequent imaging (for 1–3 min). Although in general phase separation was obtained following a stepwise decrease of membrane tension, it was also observed when ramping up from low tension (filled triangle in Fig. 4b and Supplementary Fig. 6.

We measured tube radii between 30 and 40 nm for the protein-coated part, and >70 nm for the protein-depleted one (Fig. 4c). In addition, for a subset of conditions, we observed that the lipid and protein fluorescence along the tube was inhomogeneous, yet there were not two clearly distinct domains (grey squares in Fig. 4b. See an example in Supplementary Fig. 7). This suggests incomplete phase separation, at least on the timescale (maximum 5 min) of our experiment.

Strikingly, we also found IRSp53 clustering *in vivo* which is consistent with the ability of I-BAR domain proteins to phase separate *in vitro*. Live-cell imaging experiments on U2OS cells expressing GFP-fusion of full-length IRSp53 revealed that filopodia formation was often preceded by formation of an IRSp53 cluster at the membrane. We note that similar clustering of I-BAR domain proteins IRSp53 and missing-in-metastasis (MIM) has also been reported before filopodia elongation in mouse embryonic fibroblasts and in rat primary neurons^15^,^52^. Importantly, the clustering of IRSp53 appears to be independent of actin polymerization. This is because also the isolated I-BAR domain of IRSp53, which lacks the regions required for interactions with G-actin and regulators of actin dynamics such as cdc42, VASP and Eps8 (ref. 22), is able to form clusters at the plasma membrane before the elongation of the filopodium (Supplementary Fig. 8).

Interestingly, phase separation along the tube is predicted by our model for low tensions. Briefly, it occurs because of the non-linear interaction between the density of curved proteins and tube curvature, as contained in the energy, equation (1). In fact, for a certain range of *φ*_v_ and at low tension, the tube radius is a multi-valued function of *σ*_v_ (Supplementary Note 1): one solution corresponds to a bare-like tube (*b*) in which the curvature tends to zero for vanishing tension, while the second solution (the ‘covered phase', *c*) has a finite curvature for zero tension. For the *b* and *c* phases to coexist, the axial forces in the two phases *b* and *c*, assumed to be cylindrical, must be equal and the radial forces must balance:


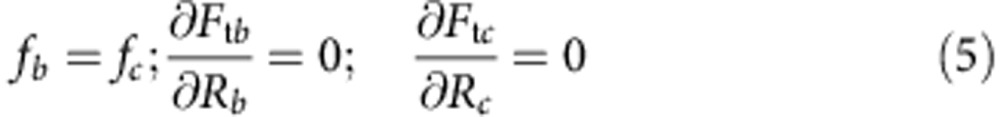


In the above, the derivative is done at constant length, lipid and protein number for a tubular domain. Solving equation (5) numerically yields the unknowns *R*_*b*_, *R*_*c*_ and the coexistence curve *φ*_v_(*σ*_v_) across which a first order-type transition takes place; see Fig. 4d. Whereas 1/*R*_*b*_ approximately follows the 
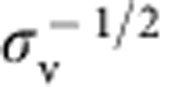
 dependence of bare tubes, 1/*R*_*c*_ weakly depends on tension, suggesting a scaffold-like protein arrangement; see inset of Fig. 4d. This predicted tension dependence of the two phases agrees qualitatively with the measurements shown in Fig. 4c.

Our model predicts that *b* and *c* domains coexist at equilibrium along the *φ*_v_(*σ*_v_) curve, as shown in Fig. 4d. However, metastable pure *b* or *c* phases occur off this curve; a thermal fluctuation may trigger nucleation of the other phase, and the resulting dynamics may be very slow in the tubular geometry. The cases of incomplete phase separation (grey squares in Fig. 4b) may be metastable states that are not completely equilibrated. The limits of metastability (spinodal curves), which can be obtained from our model (see Supplementary Note 1 for details), thus confirms that there is a window in the *φ*_v_−*σ*_v_ phase diagram in which phase-separated tubes may be observed; see Fig. 4d. The coexistence and spinodal curves converge and terminate at a critical point, shown as an asterisk (*) in Fig. 4d.

Thus, we find from our model that distinct phases, with sharply contrasting protein densities and tube radii, can coexist at low tensions and relatively low reservoir protein density, in agreement with our experimental results. Remarkably, the phase separation we have described depends only on the non-linear relation between membrane curvature and protein density, and not on any presumed direct interactions between the proteins themselves.

## Discussion

In this work, we have designed an original assay that allows studying binding of proteins on the ‘cytosolic' inner leaflet of a GUV. The vesicles obtained through this method are free of oil residues inserted within their lipid bilayer. This technique can be extended in principle to other proteins that bind to membranes essentially through electrostatic interactions. By pulling tubes from GUVs encapsulating proteins, we could measure, for the first time, the preferential binding of proteins on highly negatively curved membranes as well as the membrane deformations they induce. With this assay, we have demonstrated the strong coupling between the I-BAR domain of IRSp53 and negative membrane curvature. We have developed a general theoretical model that reproduces well the ensemble of our data, namely curvature sensing, the effect of I-BAR binding on the tube radius and on the tube force, and phase separation along the tube. Our model contains only two protein-membrane parameters, the intrinsic curvature of the protein 
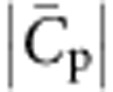
 and the elastic constant 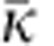. In contrast with former models, no *a priori* scaffolding is assumed^30^ or protein–protein interactions^26^,^53^ included. In principle, this model should apply to any protein that induces membrane spontaneous curvature; it can be adapted to include interactions that become significant at higher densities. In this respect, this work on IRSp53 provides a representative case study of interactions between curved proteins and curved membranes.

We have shown that IRSp53 I-BAR is maximally sorted at a curvature 
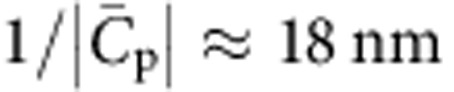
. A levelling-off of protein sorting at practically accessible curvature was already reported for a trans-membrane protein^31^ with moderate spontaneous curvature (
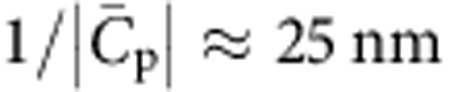
)^31^. In contrast, in the case of N-BAR domain, a maximum was not observed due to their much higher intrinsic curvature (an average of 
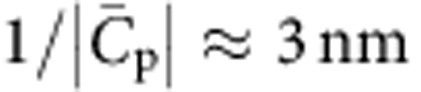
 was found for amphiphysin^26^; simulations found 
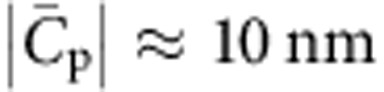
 for this protein from the membrane curvature that minimizes the binding energy^54^). Despite there being an optimum sorting at around 20 nm, we would still expect a significant curvature-driven protein enrichment in tubular structures, such as filopodia, for diameters of the order of 100 nm.

We found that IRSp53 I-BAR has significant mechanical effects on curved membranes. Even at only 5% protein coverage on the GUV, the tube radius becomes weakly sensitive to the applied tension, suggesting a scaffold-like protein arrangement on the tube^55^,^56^. Scaffolding by BAR domain proteins was previously attributed to the formation of organized protein coats, connected through either amphipathic helices (in the case of N-BAR domains^56^), or lateral interactions between adjacent BAR domains (in the case of F-BAR domains^55^). However despite its lack of amphipathic helices, and even if bound at non-saturating densities on tubes (maximum 35%), we find that the I-BAR domain of IRSp53 is able to scaffold membranes. This suggests that coupling with membrane curvature might be sufficient to explain scaffolding, in the absence of extensive protein–protein interactions.

We note here that the so-called scaffold radius, deduced from invagination assays and measured with electron microscopy, is not simply given by 
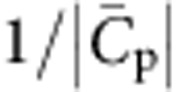
. Nonetheless, this parameter is biologically relevant, as our model predicts that the radius of a spontaneous invagination (formed at zero force) is of the order of, but slightly greater than, 
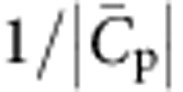
 (see Supplementary Note 1 and Supplementary Equation 32); indeed, I-BAR-induced invaginations of synthetic liposomes have an average radius of ∼20 nm (ref. 9). Biologically, IRSp53 acts in concert with other proteins and with actin bundles to form and maintain filopodia. Still, its ability to spontaneously evaginate membranes likely facilitates filopodia formation. Indeed, when overexpressed, IRSp53 can form filopodial structures, even without actin bundles^21^,^25^.

We have also shown here that the strong influence that the I-BAR has on the tube radius is mirrored by the existence of phase separation along the tube. While previously conjectured^53^, we report here the first known observation of protein phase separation on membrane tubes. Both experiments and model demonstrate that, at low tension and at low protein density, protein-covered and thin tubular domains (*c*) coexist with quasi-bare, wide ones (*b*). The radii of the *c* domains only weakly depend on tension (Fig. 4c,d), while the *b* radii follow the 
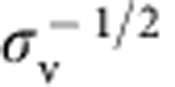
dependence of bare tubes. Interestingly, we found that direct attractive interactions between proteins are not essential for phase separation; here, indirect interactions mediated by the curved membrane tube are sufficient. This is in contrast to ref. 53, in which only direct interactions were considered. Direct interactions, which can be modelled using a mean-field expression of the type *χφ*^2^, with *χ*<0, only qualitatively shift the phase coexistence line, shown in Fig. 4d; see, also, Supplementary Fig. 9. Experimentally, there is no evidence of direct interactions between IRSp53 proteins leading to phase separation: (i) gel filtration with purified IRSp53 did not reveal any trace of oligomerization, limited amounts of oligomers were detected on a native PAGE gel (Supplementary Fig. 10), and (ii) we never optically detected IRSp53 clusters on the nearly flat GUV. These facts indicate that attractive interactions, in the absence of membrane curvature, are weak. Thus, the non-linear coupling between curvature and protein density embodied in the tube energy provides a robust mechanism for protein aggregation on membranes.

We note that incompletely decorated membrane tubes have also been seen in studies of the GTPase dynamin, which plays a role in severing membrane buds during endocytosis. In that case, however, partial coverage occurs by nucleation and growth eventually leading to the full coverage of the tube^30^. Interestingly, phase separation was not observed in a recent *in vitro* study on the N-BAR protein Amphiphysin 1 (ref. 26). The way this protein interacts with curved membranes appears similar to IRSp53; however, Amphiphysin 1 has a much greater intrinsic curvature 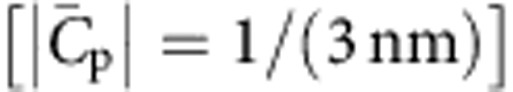, which would explain why the region of the (*φ*_v_, *σ*_v_) diagram where coexistence can occur is non-observable. Indeed, the value of the GUV protein density at the critical point in the (*φ*_v_, *σ*_v_) space is approximately given by 
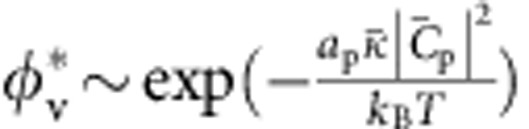
; see Supplementary Note 1. Taking *a*_p_=50 nm^2^, 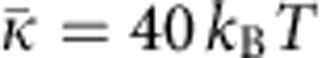 and 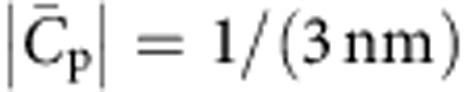, the exponential factor is completely negligible. Thus, it appears that a shallow intrinsic protein curvature is essential to observe phase separation.

**Figure 5:**
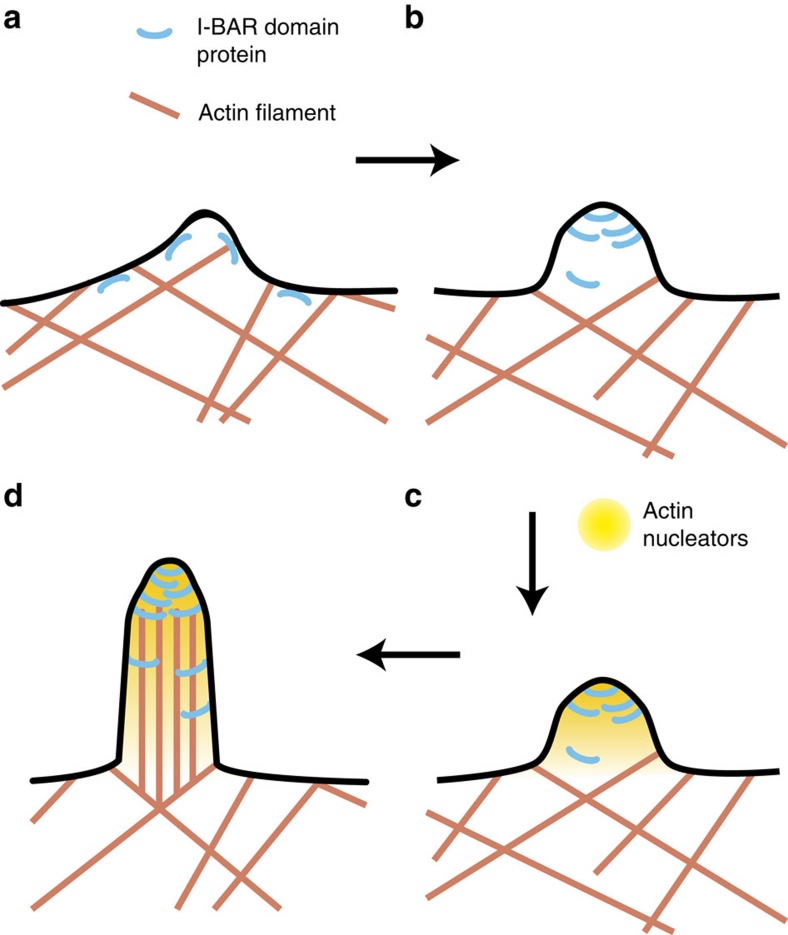
Proposed model for the role of IRSp53 in the initiation of filopodia. (**a**) I-BAR domain proteins bind to plasma membrane. The protein's intrinsic curvature favours weak negative curvature, which in turn recruits more protein. (**b**) I-BAR domain protein phase separates on curved membrane, forming an I-BAR-rich cluster at the tip of the incipient filopodium. (**c**) The PIP2 patch associated to the I-BAR cluster recruits actin polymerization nucleators. (**d**) Filopodium extension following polymerization of actin. Positive feedback between membrane deformation induced by actin polymerization and actin nucleator recruitment can additionally help filopodium growth.

We can thus propose a new mechanism for the initiation by IRSp53 of plasma membrane protrusions such as filopodia (Fig. 5). At endogenous level, active IRSp53 appears to be present at low density at the plasma membrane^15^,^21^. It was shown by Disanza *et al*.^15^ that IRSp53 forms small clusters about 1 s before recruitment of VASP at the same spot, followed by filopodium growth at the same location a few seconds later. We have also shown here that filopodia formation is often preceded by clustering of a membrane-bound IRSp53, and concomitant curving of the underlying membrane. Similar clustering was recently also reported for full-length MIM (ref. 52), suggesting that this may be a general feature of I-BAR domain proteins. Based on the ability of the I-BAR domain to be locally enriched and constrict a weakly curved membrane, a local and transient fluctuation of the membrane produced by the cytoskeleton could be stabilized and amplified^64^ through a phase separation process. The IRSp53 cluster at the tip of the deformation is expected to produce a PI(4,5)P_2_-rich domain^57^,^58^ that helps recruiting Cdc42; it also gathers actin-related proteins such as VASP, which eventually leads to the growth of filopodia^20^. The shallow curvature of the I-BAR domain is essential for this clustering to occur. Since this phase separation process is general, we could expect that it is also used by other shallow BAR domain proteins that are present at low level at the plasma membrane. This might shed light on the role of F-BAR proteins in the formation of filopodia-like structures *in vitro*^59^ and in the initiation *in vivo* of clathrin coats or of dendritic spines in neurons since these events are primed by the clustering of this type of proteins^60^,^61^.

## Methods

### Reagents

Alexa Fluor 488 C_5_-Maleimide (Alexa 488), BODIPY TR Ceramide (BODIPY TR), β-BODIPY FL C_5_-HPC (BODIPY FL) were purchased from Life Technologies. L-α-phosphatidylcholine from chicken egg (egg PC), PI(4,5)P_2_ from porcine brain, cholesterol from ovine wool, 1,2-dioleoyl-sn-glycero-3-phosphoethanolamine (DOPE), DOPS, 1,2-dioleoyl-sn-glycero-3-phosphocholine (DOPC) and 1,2-distearoyl-sn-glycero-3-phosphoethanolamine-N-(biotinyl(polyethylene glycol)-2,000; DSPE-PEG(2,000) biotin) were purchased from Avanti Polar Lipids. Streptavidin-coated polystyrene particles were from Spherotech. β-Casein (>98% pure) from bovine milk was purchased from Sigma-Aldrich.

### Plasmids

The LifeAct-RFP construct was a kind gift from Roland Wedlich-Söldner (Max-Planck Institute of Biochemistry, Martinsried, Germany. Human long isoform IRSp53 was from Japanese Collection of Research Bioresources (JCRB) and cloned into pEGFP-N1 vector (CLONTECH Laboratories, Inc.) by EcoRI-BamHI sites. GFP fusion of IRSp53 BAR domain was constructed into the XhoI–BamHI sites of pEGFP-N1.

### Purification and fluorescent labelling

The IRSp53 I-BAR domain was purified as in ref. 9. For labelling, Alexa 488 C_5_-Maleimide was dissolved at 20 mM in dimethyl sulphoxide and added at a 1:1 molar ratio to the purified protein. The mixture was kept for 30–60 mn at 4 °C under agitation. Excess dye was removed using a desalting column with 20 mM HEPES pH 7.5, 300 mM NaCl. The concentration of the protein was estimated by measuring the sample absorbance at 280 nm (with a NanoDrop ND 1,000 spectrophotometer, Thermo Scientific), and the labelling efficiency was assessed by measuring the absorbance at 488 nm. The typical labelling efficiency n* was in the range 1–2 molecules of Alexa 488 per I-BAR dimer. The sample was aliquoted, flash frozen in liquid nitrogen and stored at −80 °C until use.

### Cell culture and transfections

Human osteosarcoma (U2OS) cells (from ATCC) were cultured at +37 °C in DMEM supplemented with 10% foetal bovine serum (Hyclone), 2 mM L-glutamine, penicillin and streptomycin (Sigma-Aldrich). Transient transfection of U2OS cells was performed with FuGENE HD (Roche) according to manufacturer's instruction. Cells were re-plated on fibronectin-coated (10 μg ml^−1^ fibronectin) MatTek dishes after 24 h transfection.

### Preparation of GUVs

Lipids in powder form were solubilized in chloroform or a mixture of chloroform and methanol to prepare stock solutions. They were subsequently mixed at the following molecular ratio: 57% Egg PC, 8% PI(4,5)P_2_, 15% cholesterol, 10% DOPS, 10% DOPE, supplemented with 0.1% DSPE-PEG(2000) biotin and 0.5% BODIPY TR (for all experiments except green fluorescence calibration, see below). GUVs were prepared using an electroformation protocol suitable to work with physiological buffers^42^. A few μl of lipid mixture at 3 mg ml^−1^ were deposited onto platinum electrodes (Goodfellow). The lipid film was dried for at least 30 min. under high vacuum, then rehydrated in one of the following solutions: 200 nM IRSp53 I-BAR in 20 mM Tris pH 7.5, 100 mM NaCl, 350 mM sucrose, 1 mM EDTA and 1 mM EGTA (buffer I1) for the experiments with encapsulated I-BAR; 20 mM Tris pH 7.5, 50 mM NaCl, 150 mM sucrose, 1 mM EDTA and 1 mM EGTA (buffer I2) for experiments with bare GUVs; 20 mM Tris pH 7.5, 100 mM NaCl, 200 mM sucrose, 1 mM EDTA and 1 mM EGTA (buffer I3) for experiments with injected I-BAR. The GUVs were grown overnight at 4 °C under a sine voltage of 0.35 V RMS, 500 Hz.

### Tube experiments

Our set-up has been previously described^28^. It comprises a Nikon C1 confocal microscope equipped with optical tweezers and micromanipulators. Micropipettes were used to manipulate individual GUVs and control their tension^62^. The tension is given by 
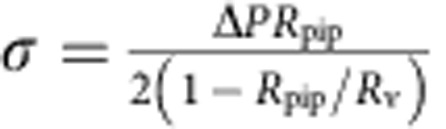
, where Δ*P* is the aspiration pressure, *R*_pip_ is the pipette radius and *R*_v_ is the vesicle radius.

Before the experiment, the chamber was filled with a solution of β-Casein at 5 mg ml^−1^, to prevent adhesion of the vesicles to the glass surface. The micropipette was back-filled with the same solution before being inserted into the experimental chamber. After a few minutes, the β-Casein solution in the chamber was replaced with the experiment buffer: 20 mM Tris pH 7.5, 300 mM NaCl, 1 mM EDTA and 1 mM EGTA (buffer O1) for the experiments with encapsulated I-BAR; 20 mM Tris pH 7.5, 100 mM NaCl, 50 mM glucose, 1 mM EDTA and 1 mM EGTA (buffer O2) for experiments with bare GUVs; 20 mM Tris pH 7.5, 200 mM NaCl, 1 mM EDTA and 1 mM EGTA (buffer O3) for experiments with injected I-BAR.

A few μl of GUVs and streptavidin-coated beads were then added to the solution. After a few minutes, both edges of the chamber were sealed with mineral oil to maintain a constant pressure inside the chamber. Finally, a GUV was aspirated in the micropipette, and a bead trapped in the optical tweezers. The GUV, held under low tension, was brought into contact with the bead, then moved away to form a membrane tube (thanks to the attachment between biotinylated lipids and streptavidin molecules at the surface of the bead). A few successive step-increases of tension were performed, with 1.5–2 min. equilibration periods. At each step, the position of the bead over time was recorded, and one fluorescence image was acquired (at the end of the 2-min period). When possible, these steps were reversed until reaching a minimal tension again.

*Injection*. For protein injection, about 1 μl of protein solution at 2 μM was aspirated into the tip of a second micropipette, and then the rest of the pipette was back-filled with mineral oil. This injection pipette was inserted into the side opposite to the first micropipette before closing the chamber. The pipette was kept high enough in the experiment chamber so that the protein would not bind to the GUVs present at the surface of the chamber. Only once a tube was pulled, the pipette was brought close to the GUV, and the measurement was performed as described above. See^26^ for more details.

### Force measurements

The force was deduced from the displacement of the bead within the trap using the expression: 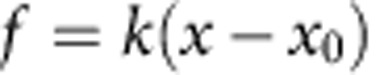 where *k* is the trap stiffness, *x* is the average position of the bead at a given tension and *x*_0_ is its equilibrium position. The position of the bead was measured by video tracking of bright field images using custom Matlab software. The trap stiffness was measured by the viscous drag method^63^.

### Image analysis

The system was imaged by fluorescence confocal microscopy in two channels corresponding to the absorption of Alexa 488 and BODIPY TR. On each image, the fluorescence intensities of the GUV and of the tube were measured using custom Matlab software. When performing the experiment, the tube was positioned parallel to the scan lines of the confocal. The fluorescence intensity was subsequently measured as the average fluorescence of the brightest line within a user-defined region of interest (ROI) including the tube. For the GUV, the ROI was positioned such that the part of the GUV analysed would be approximately parallel to the scan lines (with a small deviation due to the curvature of the GUV on confocal sections). The length of the ROI was chosen to be equal to half the radius of the vesicle.

*Measurement of tube radius*. The values of tube radius given in this article were deduced from the fluorescence intensity of the tube in the lipid (BODIPY TR) channel. The fluorescence intensity was first calibrated as previously described^26^,^31^. Briefly, for bare GUVs (in the absence of protein bound to the membrane), the radius of the tube can be calculated from the expression: 
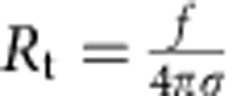
 (ref. 51), with σ the membrane tension and *f* the force measured at this tension. On the other hand, the radius is proportional to the amount of fluorophore molecules in the tube, hence to the fluorescence intensity of the tube in the lipid channel 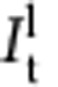: 
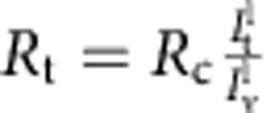
, where *R*_c_ is a constant and 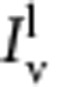 the fluorescence intensity on the GUV. The normalization is meant to cancel out variations in tube fluorescence due to an uneven distribution of the label among GUVs, or variability in the fraction of the label in the initial lipid mix. This expression holds including when proteins are bound to the membrane. Measurements of force and fluorescence were performed using bare GUVs. The quantity 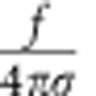 was plotted against the corresponding value of 
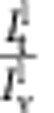
 and fitted with a linear function, yielding the calibration constant *R*_c_. We find *R*_c_=203±16 nm (*N*=11 GUVs, see Supplementary Fig. 11), in good agreement with previously reported values^26^,^31^.

*Measurement of protein density*. The density of protein bound to the membrane (number of proteins per unit area) is proportional to the fluorescence intensity in the Alexa 488 channel. The measurement of the calibration factor has also been described in refs 26, 31. Briefly, a proportionality constant *A* was first measured for the lipidated dye BODIPY FL, which can be imaged under the same settings as Alexa 488. This dye was incorporated at several molar ratios in pure DOPC vesicles, and the resulting density of dye molecules was plotted against the fluorescence intensity of the GUVs (40–50 per condition), and fitted with a linear function. A correction factor *F* accounting for the different spectral characteristics of Alexa 488 and bodipy FL was then measured: bulk solutions of both dyes were imaged, and the correction factor was taken as the ratio of their fluorescence. Finally, the density of protein bound to the membrane was calculated from the formula:
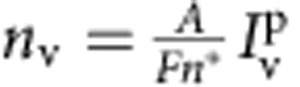
,

where n* is the labelling efficiency defined earlier.

The protein surface fraction on the GUV membrane *φ*_v_ is simply related to its density by: *φ*_v_=*a*_p_*n*_v_,

where *a*_*p*_ is the area of a single protein, 
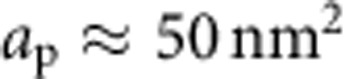
 (ref. 7).

Each GUV was characterized by its own protein density, and the full set of data was binned into three ranges of densities:

Range 1: 140≤*n*_v_≤23 μm^−2^, that is, 0.7≤*φ*_v_≤1.15%.

Range 2: 300≤*n*_v_≤500 μm^−2^, that is, 1.5≤*φ*_v_≤2.5%.

Range 3: 750≤*n*_v_≤1250 μm^−2^, that is, 3.75≤*φ*_v_≤6.25%.

*Sorting Ratio*. The sorting ratio *S* quantifies the distribution of proteins between the GUV and the tube. It is defined as 
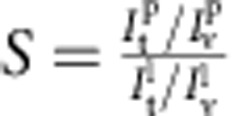
 where the ratio of green fluorescence in the tube and in the vesicle is normalized by the same ratio for red fluorescence. This is to correct for the difference in membrane areas considered in both measurements (of the tube and of the vesicle). With this definition, *S*>1 means that the protein is enriched in the tube, and *S*<1 means that it is depleted from the tube (with respect to its density on the GUV).

### Live-cell imaging

Live cells coexpressing IRSp53-GFP full-length protein or isolated BAR domain together with LifeAct-RFP were imaged in 37 °C under a CO2 hood with 3I Marianas (3I intelligent Imaging Innovations). All images were acquired by Zeiss Axio Observer Z1 microsope using a C-Apochromat × 63/1.2 numerical aperture water objective and 488/561 nm solid-state lasers modulated at 50 MHz. 488 s/561 s filters were used for live-cell imaging and images was taken every 2 s. Image analysis was performed using ImageJ software.

## Additional information

**How to cite this article:** Prévost, C. *et al*. IRSp53 senses negative membrane curvature and phase separates along membrane tubules. *Nat. Commun.* 6:8529 doi: 10.1038/ncomms9529 (2015).

## Supplementary Material

Supplementary Information Supplementary Figures 1-11, Supplementary Note 1 and Supplementary References
