# Supplementary material for: IRSp53 senses negative membrane curvature and phase separates along membrane tubules
Source: Nat Commun. 2015 Oct 15;6:8529. doi: 10.1038/ncomms9529 (PMC4634128; doi:10.1038/ncomms9529)
Supplement: Supplementary Information — Supplementary Figures 1-11, Supplementary Note 1 and Supplementary References [file ncomms9529-s1.pdf]

Supplementary Figures.

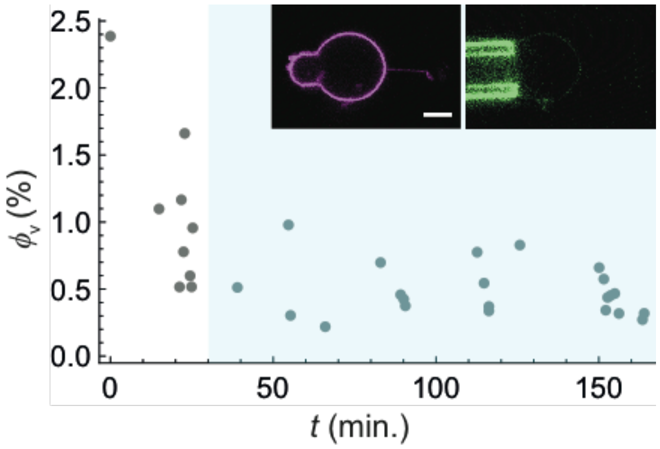

Supplementary Figure 1: Desorption of proteins bound to the external leaflet of GUV and tube in the presence of 300 mM NaCl buffer. GUVs were grown in buffer I1 (see Methods) in the absence of protein. They were subsequently incubated for 3h with 100 nM I-BAR domain in the same buffer. A few µL of GUVs were then added to the experiment chamber filled with the external buffer containing 300 mM NaCl (buffer O1). 31 images of GUVs were recorded at different times to monitor the evolution of the protein area fraction on their external leaflet (the first image was recorded ~ 1 min. after injection of the GUVs in the buffer O1). In a typical experiment with encapsulated I-BAR, measurements were started ~ 30 min after adding the GUVs to the chamber (which corresponds to the blue zone of the plot). For conditions corresponding to the blue zone, the mean protein area fraction left on the GUVs is ~ 0.5%. In addition, there is no detectable protein binding on tubes pulled from these GUVs under our imaging conditions (see a representative image in inset. N=3 GUVs). It shows that proteins remaining on the external leaflet contribute only marginally to fluorescence measurements. In practice this means that we might overestimate ϕ_v by about 0.5%, while the uncertainty on ϕ_t is negligible. Scale bar, 5µm.

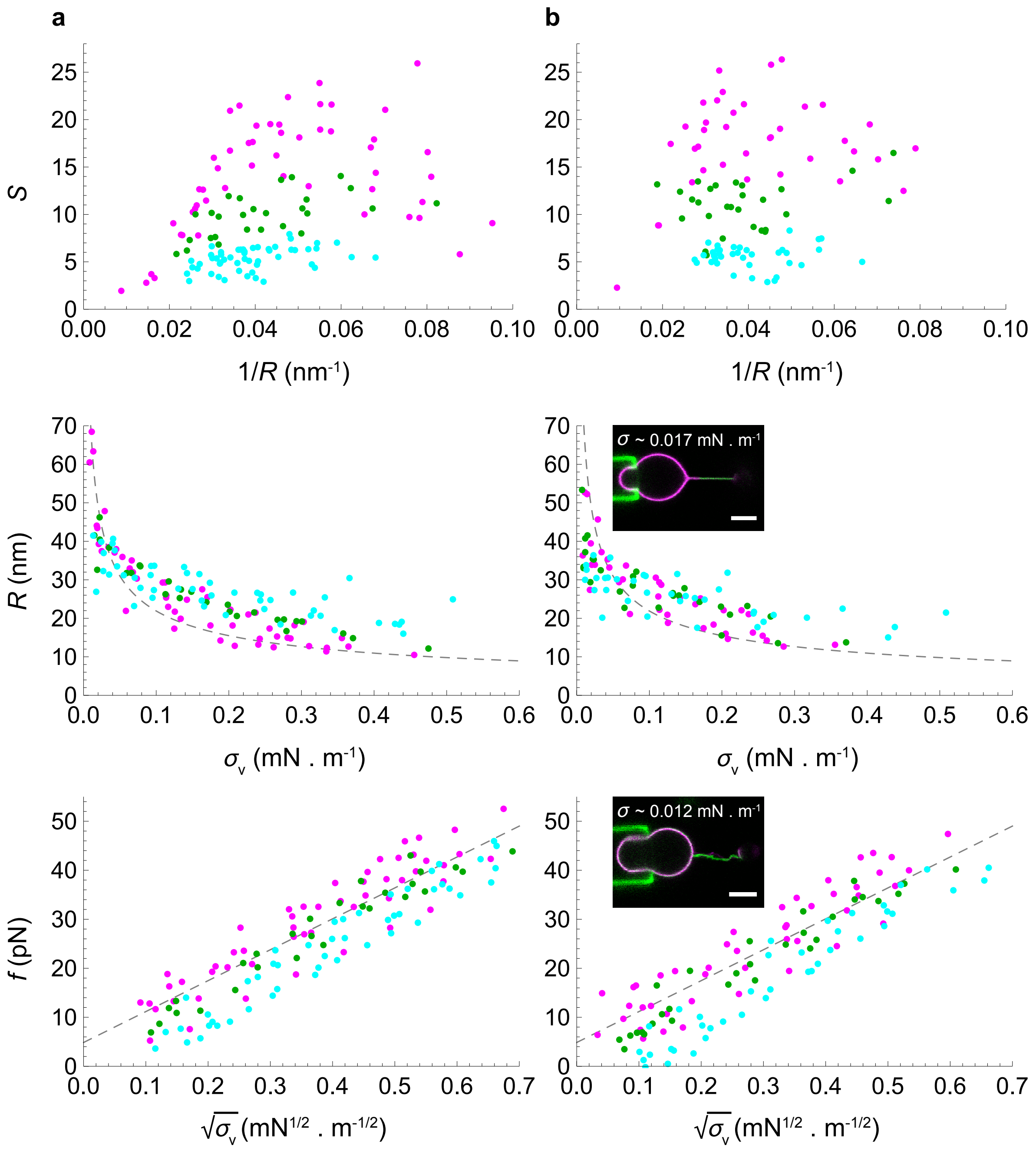

Supplementary Figure 2: Reversibility of the measurements. (a) Data collected upon stepwise increase of membrane tension. Magenta: $\phi_v \approx 1\%$, N=9 GUVs; green: $\phi_v \approx 2\%$, N=5 GUVs; cyan: $\phi_v \approx 5\%$, N=10 GUVs. (b) Data collected upon stepwise decrease of membrane tension. Same color code. Magenta: N=8 GUVs; green: N=5 GUVs; cyan: N=8 GUVs. As maintaining a stable system under very low membrane tension is particularly delicate, the measurements in this condition were put off to the end of each experiment (end of the stepwise decrease of membrane tension). In particular, the observations of floppy tubes and of phase-separated tubes were made at the end of a subset of experiments (insets). Scale bar, 5 µm.

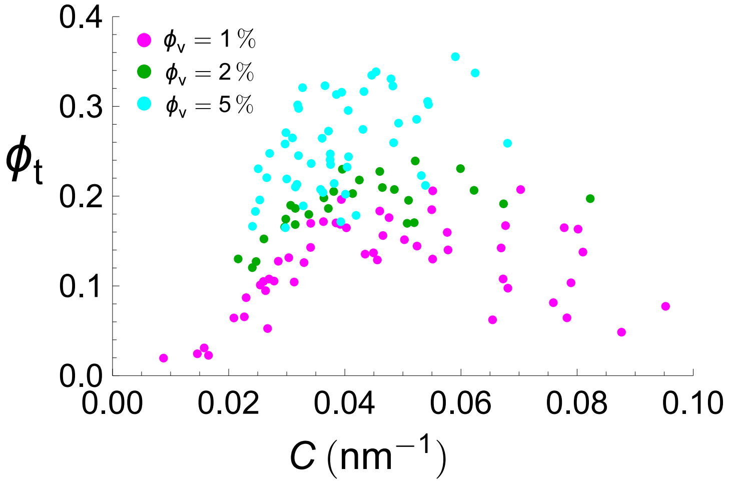

Supplementary Figure 3: The absolute density of protein on the tube ϕ_t increases with the protein density on the GUV ϕ_v. Magenta: $\phi_v \approx 1\%$, N=9 GUVs; green: $\phi_v \approx 2\%$, N=5 GUVs; cyan: $\phi_v \approx 5\%$, N=10 GUVs.

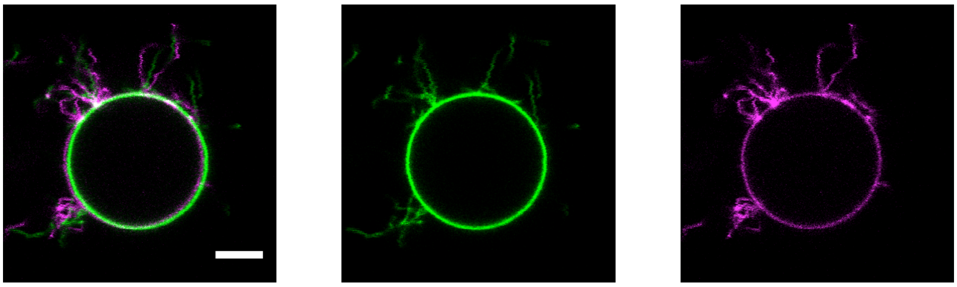

Supplementary Figure 4: Encapsulated IRSp53 I-BARs produce membrane protrusions. GUVs were grown in I1 buffer containing 100 nM IRSp53 I-BAR. A few µL of the sample was transferred to 200 µL of buffer O1. Tubules are very flexible and both channels might not overlap due to their movement. Scale bar, 5 µm.

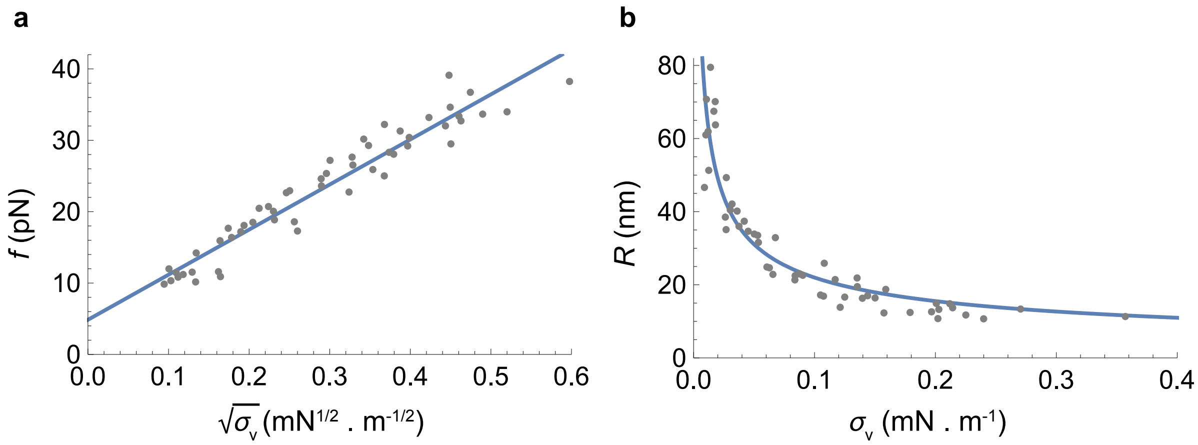

Supplementary Figure 5: Tube force and radius versus GUV tension σ_{v} for bare membranes (reference curves). Tubes were pulled from bare GUVs (grown in buffer I2, and transferred in buffer O2 for the experiment). The force and the radius at each tension step were measured as described in Methods. N=11 GUVs. (a) Force data and fit. Fitting the equation $f = f_0 + 2\pi\sqrt{2\kappa\sigma}$ to the data yields an offset $f_0 = 4.9 \pm 1.6$ pN and bending rigidity $\kappa = 13 \pm 2\ k_{\mathrm{B}}T$ (with 95% confidence bounds). (b) Radius data and fit. Fitting the equation $R = \sqrt{\frac{\kappa}{2\sigma}}$ to the data yields: $\kappa = 24 \pm 2.6\ k_{\mathrm{B}}T$ (with 95% confidence bounds).

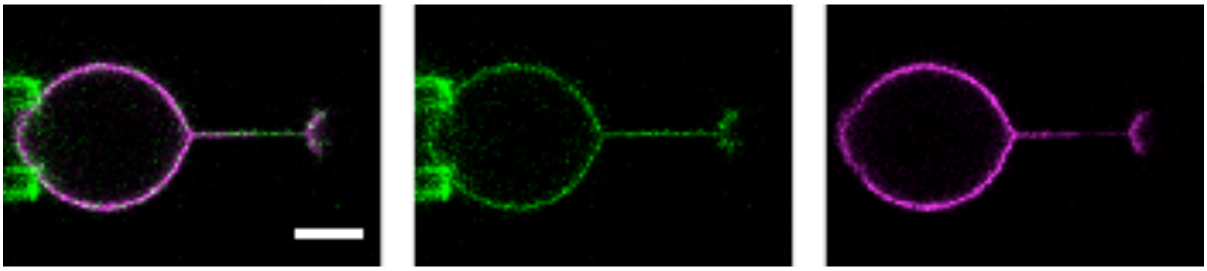

Supplementary Figure 6: Phase separation is independent of the tension cycle history. A tube was pulled from a GUV, and a low tension ($\sigma = 0.002\ \mathrm{mN\ m^{-1}}$) was applied. Phase separation was observed ~ 1 min. after the change in membrane tension, and did not evolve over the observation time (3 min). This was similar to our observations when going from high to low tension. Here $\phi_\mathrm{v} = 2.3\%$. Scale bar, 5 µm.

a

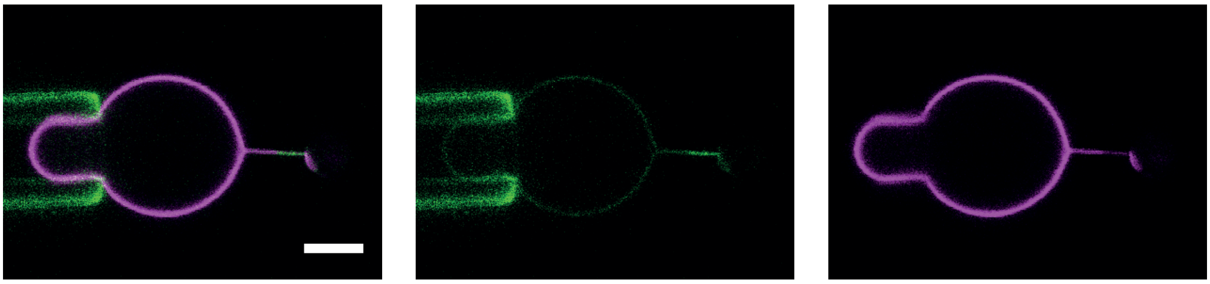

b

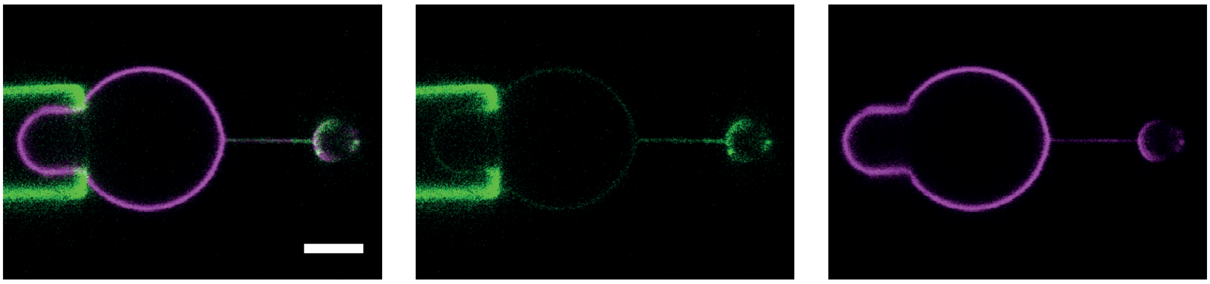

Supplementary Figure 7: "Complete" versus "incomplete" phase separation. (a) In the case of "complete phase separation", we observe two distinct domains and a sharp limit between a "covered phase" and a "quasi-bare phase". Here $\phi_v = 1.0\%$ and $\sigma = 0.01$ mN m^{-1}. (b) In the case of "incomplete phase separation", the limit between both phases is smoother and more domains are detected. $\phi_v = 0.9\%$ and $\sigma = 0.01$ mN m^{-1}. Scale bar, 5 µm.

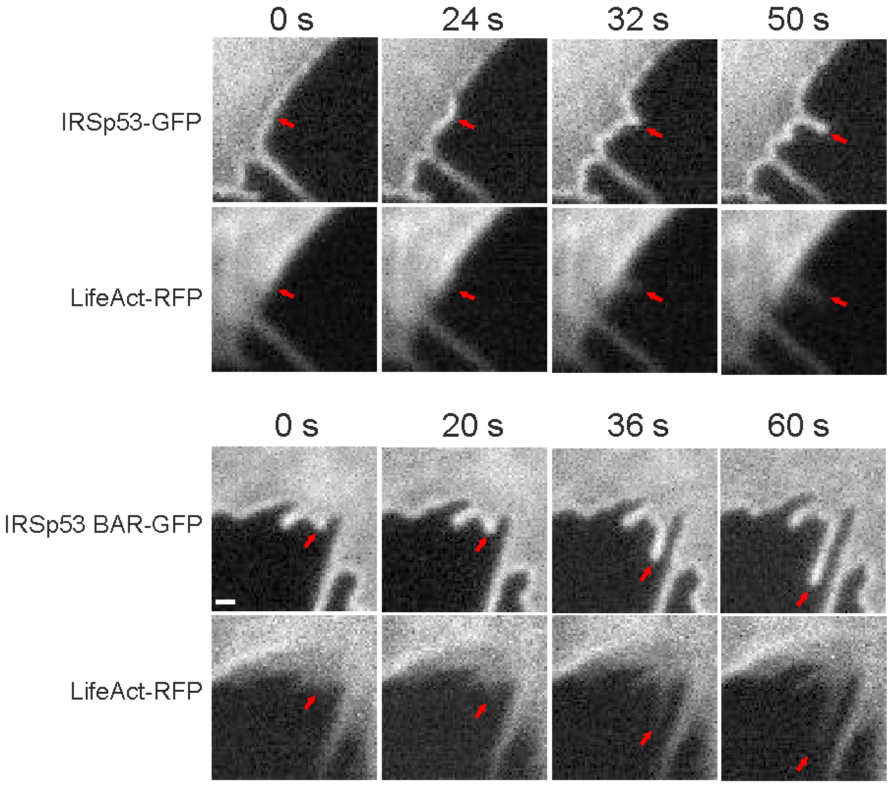

Supplementary Figure 8: IRSp53 and its isolated I-BAR domain form clusters at the plasma membrane. Time frames from U2OS cells expressing either GFP-IRSp53 and Lifeact-RFP (top panels) or GFP-fusion of the isolated I-BAR domain of IRSp53 and Lifeact-RFP (bottom panels). Note that filopodium initiation/elongation (indicated by arrowheads) is in both cases preceded by clustering of IRSp53 or its isolated I-BAR domain. Scale bar represents 1 µm.

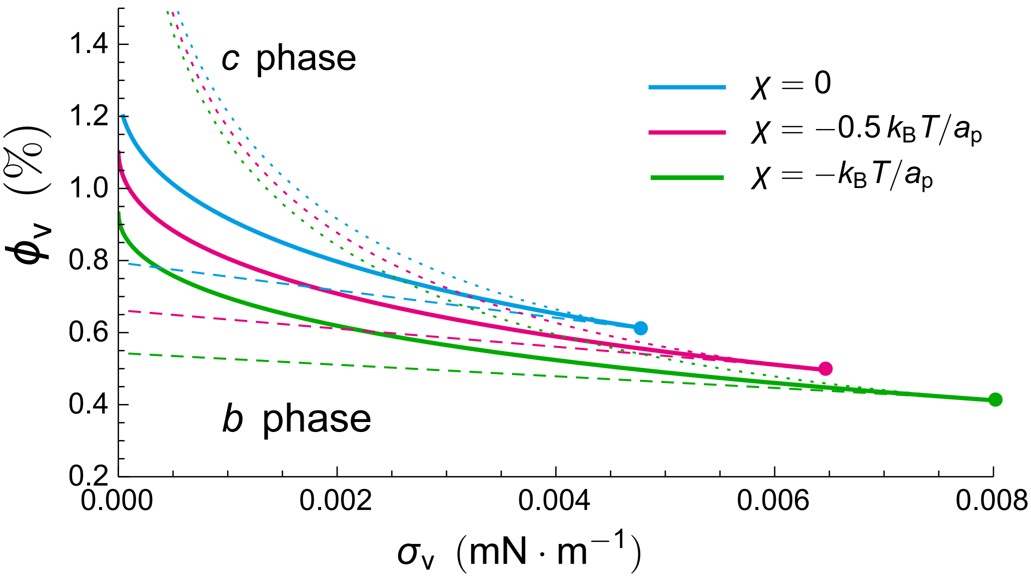

Supplementary Figure 9: Effect of protein-protein interactions on phase separation. Direct, attractive protein-protein interactions are found to shift the coexistence and spinodal curves in the $\phi_\mathrm{v} - \sigma_\mathrm{v}$ phase diagram. Curves for three values of the interaction parameter χ (see Eq. S5) are shown. As expected, they favor the covered (c) phase over the bare (b) phase at smaller values of ϕ_v than in their absence. They do not, however, qualitatively change the topology of the phase diagram. The other model parameters used in this figure are $\kappa = 24\, k_\mathrm{B}T$, $|\overline{C}_\mathrm{p}| = 0.055\,\mathrm{nm}^{-1}$, $\bar{\kappa} = 70\, k_\mathrm{B}T$, $a_\mathrm{p} = a_\mathrm{l} = 50\,\mathrm{nm}^2$.

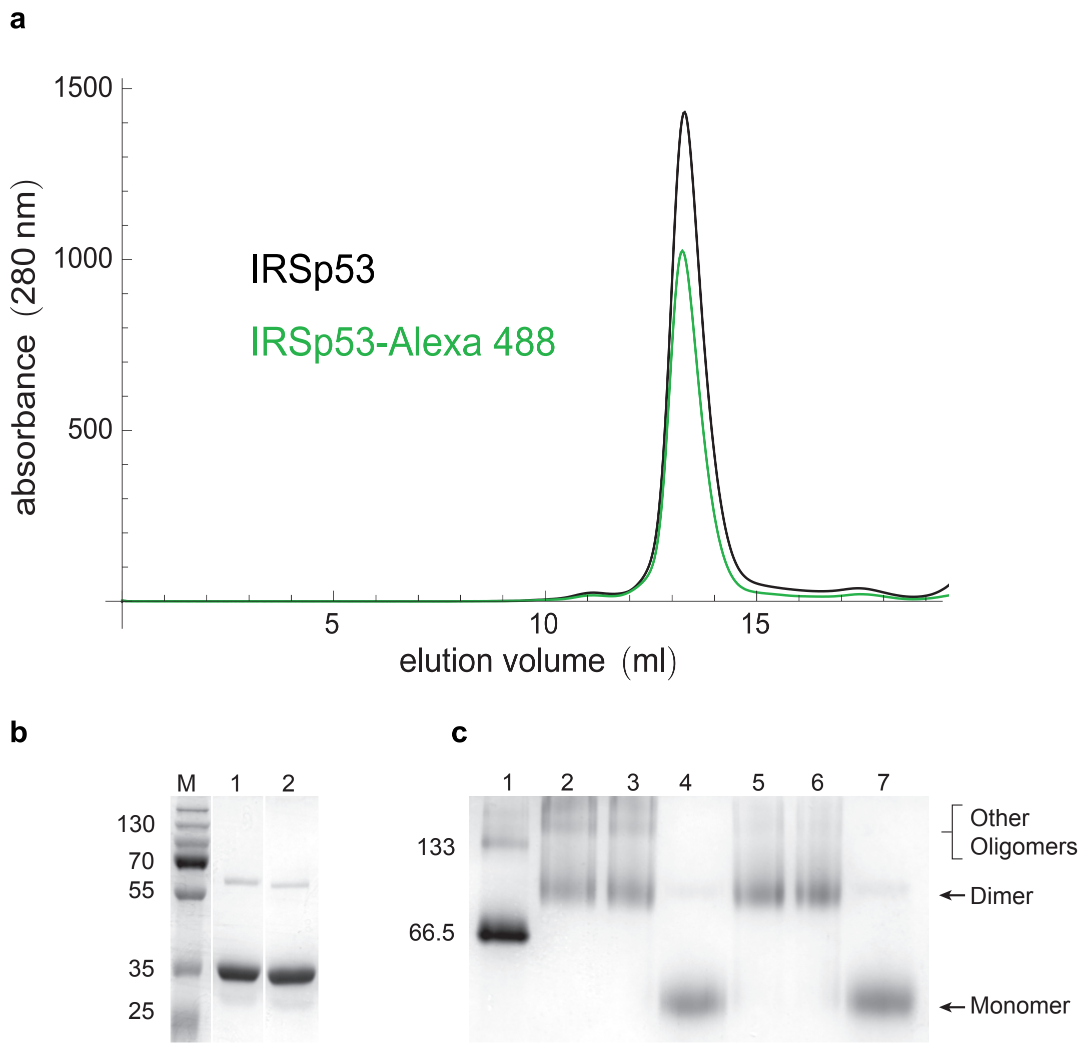

Supplementary Figure 10: Gel Filtration on IRSp53 reveals no oligomerization in bulk.

(a) Size Exclusion Chromatography Profile of purified IRSp53. IRSp53 and IRSp53-Alexa488 were run over the Superdex 200 10/300 GL column in 20 mM Tris, 250 mM NaCl Buffer. The chromatogram indicates one clear peak at 13.29 ml and 13.24 ml, respectively, indicating IRSp53 is in fact a dimer.

(b) SDS-PAGE of purified IRSp53. The IRSp53 peak from (a) was run on a 12% SDS-PAGE Gel and stained with Coomassie Blue. Lane M: Markers. *Lane 1*: IRSp53-Alexa488, *Lane 2*: IRSp53. Under these conditions, IRSp53 runs as a monomer and has an approximate molecular weight of 35 kDa.

(c) Native-PAGE of purified IRSp53. 3 µg of the IRSp53 peak from (a) was run on a 12% Native-PAGE Gel and stained with Coomassie Blue. Lane 1: BSA standard. Lanes 2-4: IRSp53 and Lanes 5-7: IRSp53-Alexa488. Lanes 2 and 5 were treated with purification

buffer (20 mM Tris, 250 mM NaCl) and Lanes 3 and 6 were treated with experimental buffer (20mM Tris pH 7,5, 100 mM NaCl, 1mM EDTA). In both conditions, IRSp53 is mainly in its dimeric form with other possible oligomers present. Lanes 4 and 7 were treated with SDS. IRSp53 is denatured and forms monomers.

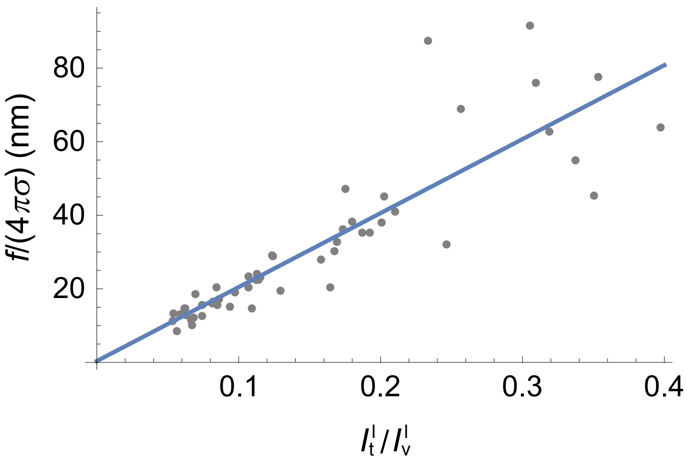

Supplementary Figure 11: Calibration of the tube radius. Determination of the proportionality constant between the radius of the tube and its fluorescence. The experimental procedure is the same as described in Supplementary Figure 5. N=11 GUVs. The data were fitted with a linear function, yielding the calibration constant R_c: R_c =203 +/-16 nm.

Supplementary Note 1

We present here the mathematical description of membrane tubes decorated with IRSp53 I-BAR proteins. We first write down the membrane free energy in the presence of bound IRSp53. We then calculate the enrichment of protein on the tube relative to the GUV at equilibrium, the force on the tube, and the tube radius. Finally, we show that our model predicts phase separation at low tensions, in agreement with experiments.

1 Energy of membrane with bound protein

In the presence of IRSp53 bound to the inner leaflet, the membrane free energy consists of four terms. Below, we write these contributions in a generic form that can be applied to the tube or to the GUV:

1. *Phospholipid membrane bending energy.* Assuming constant mean curvature, H, and neglecting spontaneous curvature this is given by

$$F_{\mathrm{b}} = A\,\frac{\kappa}{2}\,(2H)^2\ . \qquad \text{S1}$$

   In this equation, A is either the area of the GUV vesicle ($A = A_{\mathrm{v}}$) or the area of the tube ($A = A_{\mathrm{t}}$). In the case of the vesicle, the mean curvature is $H \approx 0$; for the tube, $H \approx 1/(2R)$, R being the tube radius.

2. *Protein-membrane interaction.* Protein binding to the membrane involves a combination of protein and local membrane deformation. To lowest order in protein density, the interaction energy is proportional to protein number and proportional to the difference between membrane and intrinsic protein curvature:

$$F_{\mathrm{int}} = A\,\left(\frac{1}{2}\overline{\kappa}\,a_{\mathrm{p}}\,n_{\mathrm{p}}\,\left(2H - \overline{C}_{\mathrm{p}}\right)^2\right)\ . \qquad \text{S2}$$

   In this equation $\overline{\kappa}$ is an elastic constant reflecting the energy penalty for mismatch been protein and membrane and curvatures. Also, a_{p} is the area of a single protein, n_{p} is the protein number density (per unit area), and $\overline{C}_{\mathrm{p}}$ is a phenomenological coefficient related to the protein's intrinsic curvature. We note that the interaction energy above is very closely related to the spontaneous curvature model proposed in Ref. [1]. There are two differences. First, we now include a higher-order curvature term, $\sim n_{\mathrm{p}}\,H^2$, that is necessary to account for the observed sorting maximum for IRSp53. Second, we discard protein-protein terms $\sim n_{\mathrm{p}}^2$, since our current approach is to emphasize the importance of protein-membrane interactions in driving tube mechanical changes and protein phase separation.

3. *In-plane membrane stretching energy.* The stretching energy (related to the membrane tension) is given by

$$F_{\mathrm{s}} = A\, f_{\mathrm{s}}(T, n_{\mathrm{l}}, n_{\mathrm{p}})\,, \qquad \text{S3}$$

where as a convenient model choice for the density f_{s} we take

$$f_{\mathrm{s}}(T, n_{\mathrm{l}}, n_{\mathrm{p}}) = \frac{1}{2} k \left(n_{\mathrm{l}}\, a_{\mathrm{l}} + n_{\mathrm{p}}\, a_{\mathrm{p}} - 1\right)^2\,. \qquad \text{S4}$$

Here, k is the compression/dilatation modulus, n_{l} is the lipid number density, and a_{l} is the area per lipid. The quantity in parentheses above is the relative change in area of a membrane patch containing lipids and proteins, going from initial area a_0 to final area a: $n_{\mathrm{l}}\, a_{\mathrm{l}} + n_{\mathrm{p}}\, a_{\mathrm{p}} - 1 = a_0/a - 1$. We remark that after calculating equilibrium intensive variables (chemical potentials, membrane tension), we will take the incompressible membrane limit, $k \to \infty$, so that our particular choice of f_{s} is not crucial.

4. *Mixing free energy.* Inhomogeneities in a two component membrane (lipids plus proteins) is entropically unfavorable. Accounting for the differences in lipid and protein molecular areas and including direct protein-protein interactions, the mixing free energy is $F_{\mathrm{m}} = A\, f_{\mathrm{m}}$, with the density f_{m} is taken to have the Flory-Huggins form:

$$f_{\mathrm{m}}(T, n_{\mathrm{l}}, n_{\mathrm{p}}) = k_{\mathrm{B}} T \left[n_{\mathrm{l}} \ln\left(\frac{a_{\mathrm{l}}\, n_{\mathrm{l}}}{a_{\mathrm{l}}\, n_{\mathrm{l}} + a_{\mathrm{p}}\, n_{\mathrm{p}}}\right) + n_{\mathrm{p}} \ln\left(\frac{a_{\mathrm{p}}\, n_{\mathrm{p}}}{a_{\mathrm{l}}\, n_{\mathrm{l}} + a_{\mathrm{p}}\, n_{\mathrm{p}}}\right) + \frac{\chi}{a_{\mathrm{p}}} n_{\mathrm{p}}^2 \right]\,, \qquad \text{S5}$$

where χ is a direct interaction coefficient ($\chi < 0$ (> 0) for attractive (repulsive) interactions). In the following, we neglect interactions (setting $\chi = 0$), except at the end of Sec. 4.3. Note that the entropic part of f_{m} is zero in the cases of a pure lipid ($n_{\mathrm{p}} = 0$) or pure protein ($n_{\mathrm{l}} = 0$) membrane, as it should. The arguments of the logarithmic terms are just the protein and lipid area fractions: $\phi = \frac{a_{\mathrm{p}}\, n_{\mathrm{p}}}{a_{\mathrm{l}}\, n_{\mathrm{l}} + a_{\mathrm{p}}\, n_{\mathrm{p}}}$ and $1 - \phi = \frac{a_{\mathrm{l}}\, n_{\mathrm{l}}}{a_{\mathrm{l}}\, n_{\mathrm{l}} + a_{\mathrm{p}}\, n_{\mathrm{p}}}$.

Combining all energetic terms, and neglecting vesicle curvature its total vesicle energy is

$$F_{\mathrm{v}} = A_{\mathrm{v}}\, f_{\mathrm{v}} = A_{\mathrm{v}} \left[\frac{\overline{\kappa}}{2}\, a_{\mathrm{p}}\, n_{\mathrm{p}}^{\mathrm{v}}\, \overline{C}_{\mathrm{p}}^2 + f_{\mathrm{s}}(T, n_{\mathrm{l}}^{\mathrm{v}}, n_{\mathrm{p}}^{\mathrm{v}}) + f_{\mathrm{m}}(T, n_{\mathrm{l}}^{\mathrm{v}}, n_{\mathrm{p}}^{\mathrm{v}}) \right]\,. \qquad \text{S6}$$

where A_{v} is the vesicle area, including that of the aspirated tongue. Similarly, the tube energy is

$$F_{\mathrm{t}} = 2\pi\, R\, L\, f_{\mathrm{t}} = 2\pi\, R\, L \left[\frac{\kappa}{2} \frac{1}{R^2} + \frac{\overline{\kappa}}{2}\, a_{\mathrm{p}}\, n_{\mathrm{p}}^{\mathrm{t}} \left(\frac{1}{R} - \overline{C}_{\mathrm{p}}\right)^2 + f_{\mathrm{s}}(T, n_{\mathrm{l}}^{\mathrm{t}}, n_{\mathrm{p}}^{\mathrm{t}}) + f_{\mathrm{m}}(T, n_{\mathrm{l}}^{\mathrm{t}}, n_{\mathrm{p}}^{\mathrm{t}}) \right] \qquad \text{S7}$$

In the above equations, n^{v} and n^{t} refer to vesicle and tube densities.

2 Membrane tension

2.1 GUV tension

The vesicle tension, given thermodynamically by

$$\sigma_{\mathrm{v}} = \left(\frac{\partial F_{\mathrm{v}}}{\partial A_{\mathrm{v}}}\right)_{T,N_{\mathrm{l}},N_{\mathrm{p}}}, \tag{S8}$$

is determined as follows. First, the work done by the pressures external to the vesicle (spherical vesicle of radius R_{v} plus aspirated tongue) during an infinitesimal, virtual transformation in which the tongue volume changes by δV_{tongue} and the volume of the spherical part of the vesicle changes by $\delta V_{\text{sphere}} = -\delta V_{\text{tongue}}$ (the total vesicle volume V_{v} is assumed constant, since permeation through the membrane is neglected), is

$$\delta W = -P_{\text{pip}}\,\delta V_{\text{tongue}} - P_0\,\delta V_{\text{sphere}} = \Delta p\,\delta V_{\text{tongue}}\,. \tag{S9}$$

In this equation, P_{pip} and P_0 are the pressures external to the tongue inside the pipette and to the spherical part of the vesicle. Also, $\Delta p = P_0 - P_{\text{pip}} > 0$ is the aspiration pressure. Second, performing the virtual transformation about the equilibrium state, the work done is equal to the change in free energy:

$$\delta F_{\mathrm{v}} = \Delta p\,\delta V_{\text{tongue}}\,. \tag{S10}$$

On the other hand, $\delta F_{\mathrm{v}} = \frac{\partial F_{\mathrm{v}}}{\partial A_{\mathrm{v}}}\,\delta A_{\mathrm{v}} = \sigma_{\mathrm{v}}\,\delta A_{\mathrm{v}}$. Assuming a cylindrical tongue of constant radius, the change in A_{v} at constant V_{v} is $\delta A_{\mathrm{v}} = \frac{2}{R_{\text{tongue}}}\left(1 - \frac{R_{\text{tongue}}}{R_{\text{sphere}}}\right)\delta V_{\text{tongue}}$. As a result, the vesicle tension is

$$\sigma_{\mathrm{v}} = \frac{\Delta p\,R_{\text{tongue}}}{2\left(1 - \frac{R_{\text{tongue}}}{R_{\text{sphere}}}\right)}\,. \tag{S11}$$

Thus, the vesicle tension, defined here thermodynamically, agrees with the usual Laplace expression for aspirated GUVs [2]. We note that in the incompressible limit, $k \to \infty$, σ_{v} is calculated in terms of the lipid and protein densities, giving

$$\sigma_{\mathrm{v}} = k\left(1 - a_{\mathrm{l}}\,n_{\mathrm{l}}^{\mathrm{v}} - a_{\mathrm{p}}\,n_{\mathrm{p}}^{\mathrm{v}}\right)\,. \tag{S12}$$

Therefore, for $k \to \infty$ the lipid and protein densities are related through $a_{\mathrm{l}}\,n_{\mathrm{l}}^{\mathrm{v}} + a_{\mathrm{p}}\,n_{\mathrm{p}}^{\mathrm{v}} \simeq 1$.

2.2 Tube tension

The tension on the tube is defined through the stretching energy on the tube as

$$\sigma_{\rm t} = \left(\frac{\partial F_{\rm s,t}}{\partial A_{\rm t}}\right)_{T,N_{\rm l}^{\rm t},N_{\rm p}^{\rm t}} . \qquad \text{S13}$$

This definition is consistent with the earlier one for $\sigma_{\rm v}$ since we do not include bending and protein-membrane energies (the mixing energy does not contribute since it does not depend explicitly on area). Taking $k \to \infty$ we find

$$\sigma_{\rm t} = k\left(1 - a_{\rm l}\, n_{\rm l}^{\rm t} - a_{\rm p}\, n_{\rm p}^{\rm t}\right) . \qquad \text{S14}$$

In this limit the lipid and protein densities are related through $a_{\rm l}\, n_{\rm l}^{\rm t} + a_{\rm p}\, n_{\rm p}^{\rm t} \simeq 1$. We note that, while $\sigma_{\rm v}$ is controlled by pipette aspiration and is known, $\sigma_{\rm t}$ is not.

3 Protein sorting

The relative enrichment of protein on the tube at equilibrium is calculated by balancing lipid and protein chemical potentials on the GUV and on the tube. These are calculated below.

3.1 Chemical potentials

The lipid chemical potential is given by $\mu_{\rm l}^{\rm v} = \left(\frac{\partial F_{\rm v}}{\partial N_{\rm l}}\right)_{T,A_{\rm v},N_{\rm p}} = \left(\frac{\partial f_{\rm v}}{\partial n_{\rm l}^{\rm v}}\right)_{n_{\rm p}^{\rm v}}$. Carrying out the differentiation with respect to $n_{\rm l}^{\rm v}$and then taking $k \to \infty$,

$$\mu_{\rm l}^{\rm v} = -a_{\rm l}\, \sigma_{\rm v} + k_{\rm B}T\left[\ln\left(a_{\rm l}\, n_{\rm l}^{\rm v}\right) + n_{\rm p}^{\rm v}\, \left(a_{\rm p} - a_{\rm l}\right)\right] . \qquad \text{S15}$$

Similarly, the protein chemical potential $\mu_{\rm p}^{\rm v} = \left(\frac{\partial F_{\rm v}}{\partial N_{\rm p}}\right)_{T,A_{\rm v},N_{\rm l}} = \left(\frac{\partial f_{\rm v}}{\partial n_{\rm p}^{\rm v}}\right)_{n_{\rm l}^{\rm v}}$ is

$$\mu_{\rm p}^{\rm v} = -a_{\rm p}\, \sigma_{\rm v} + a_{\rm p}\, \frac{\overline{\kappa}}{2}\overline{C}_{\rm p}^2 + k_{\rm B}T\left[\ln\left(a_{\rm p}\, n_{\rm p}^{\rm v}\right) - n_{\rm l}^{\rm v}\, \left(a_{\rm p} - a_{\rm l}\right)\right] . \qquad \text{S16}$$

The chemical potentials on the tube are found in the same way, yielding

$$\mu_{\rm l}^{\rm t} = -a_{\rm l}\, \sigma_{\rm t} + k_{\rm B}T\left[\ln\left(a_{\rm l}\, n_{\rm l}^{\rm t}\right) + n_{\rm p}^{\rm t}\, \left(a_{\rm p} - a_{\rm l}\right)\right] , \qquad \text{S17}$$

and

$$\mu_{\rm p}^{\rm t} = -a_{\rm p}\, \sigma_{\rm t} + \frac{\overline{\kappa}}{2}\, a_{\rm p}\left(\frac{1}{R} - \overline{C}_{\rm p}\right)^2 + k_{\rm B}T\left[\ln\left(a_{\rm p}\, n_{\rm p}^{\rm t}\right) - n_{\rm l}^{\rm t}\, \left(a_{\rm p} - a_{\rm l}\right)\right] . \qquad \text{S18}$$

We note that the tube tension can be expressed in terms of the controlled tension $\sigma_{\rm v}$ using the equilibrium condition $\mu_{\rm l}^{\rm v} = \mu_{\rm l}^{\rm t}$. Using Eqs. S15 and S17, taking the incompressible limit, and introducing the protein area fractions $\phi_{\rm v} = a_{\rm p}\, n_{\rm p}^{\rm v}$ and $\phi_{\rm t} = a_{\rm p}\, n_{\rm p}^{\rm t}$, we find

$$\sigma_{\rm t} = \sigma_{\rm v} + \frac{k_{\rm B}T}{a_{\rm l}}\left[\ln\left(\frac{1-\phi_{\rm t}}{1-\phi_{\rm v}}\right) + \left(1-\frac{a_{\rm l}}{a_{\rm p}}\right)(\phi_{\rm t}-\phi_{\rm v})\right]. \qquad \text{S19}$$

In the dilute limit, $\phi_{\rm t}$, $\phi_{\rm v} \ll 1$, the above expression simplifies to $\sigma_{\rm t} \simeq \sigma_{\rm v} - k_{\rm B}T\left(n_{\rm p}^{\rm t} - n_{\rm p}^{\rm v}\right)$, which is the Gibbs expression describing the reduction of interfacial tension by an adsorbate [3].

3.2 Sorting

To find the sorting, we equate $\mu_{\rm p}^{\rm v} = \mu_{\rm p}^{\rm t}$ and use Eqs. S16 and S18. Applying incompressibility and Eq. S19, an implicit expression for the sorting $S = \phi_{\rm t}/\phi_{\rm v}$ is found:

$$S\left(\frac{1-\phi_{\rm v}}{1-\phi_{\rm v}\,S}\right)^{\gamma} = \exp\left[\frac{\overline{\kappa}\, a_{\rm p}}{k_{\rm B}T}\left(\frac{\overline{C}_{\rm p}}{R} - \frac{1}{2R^2}\right)\right], \qquad \text{S20}$$

where $\gamma = a_{\rm p}/a_{\rm l}$. In the dilute limit, $\phi_{\rm v} \to 0$, an explicit expression for S, and independent of $\phi_{\rm v}$, is obtained:

$$S \simeq \exp\left[\frac{\overline{\kappa}\, a_{\rm p}}{k_{\rm B}T}\left(\frac{\overline{C}_{\rm p}}{R} - \frac{1}{2R^2}\right)\right], \qquad \text{S21}$$

indicating that S as a function of tube curvature $C = 1/R$ is Gaussian with a maximum at $C = \overline{C}_{\rm p}$. For the general case of $\phi_{\rm v}$ not necessarily small and $a_{\rm p} \neq a_{\rm l}$, Eq. S20 must be solved numerically for $S(C)$. However, two general conclusions can be drawn analytically from Eq. S20. First, the maximum of $S(C)$ remains at $C = \overline{C}_{\rm p}$; from the experimental data, Fig. 2, we can therefore determine $\overline{C}_{\rm p}$ from the three density ranges explored. Second, a short calculation reveals that for $S > 1$ and $\gamma > 1$, for fixed tube curvature S is a decreasing function of $\phi_{\rm v}$, in agreement with experiment. Henceforth, we assume for analytical simplicity $\gamma = 1$, though taking $\gamma > 1$ does not qualitatively change our results.

4 Tube mechanics

4.1 Tube radius

The tube radius, R, at equilibrium, is found by relating the virtual work done by the pressure difference across the tube, $P_{\rm t} - P_0$, to the change in free energy of a cylindrical portion of tube, of fixed number of lipids and proteins:

$$\left(\frac{\partial F_{\rm t}}{\partial R}\right)_{L,N_{\rm l},N_{\rm p}} \delta R = (P_{\rm t} - P_0)\,\delta V_{\rm t}\,. \qquad \text{S22}$$

In the above, the length of the tube portion is held fixed, and thus the axial force, f, acting at the ends of the portion, does no work. (At equilibrium f is uniform along the tube, and given by the force exerted by the bead on the tube end). At constant L, $\delta V_{\mathrm{t}} = 2\pi\, R\, L\, \delta R$, and $P_{\mathrm{t}} - P_0$ is given by $2\sigma_{\mathrm{v}}/R_{\mathrm{sphere}}$ (since, at equilibrium, the pressure inside the tube is the same as that inside the mother vesicle). Therefore, the pressure work term above is $\sim \sigma_{\mathrm{v}} \frac{R}{R_{\mathrm{sphere}}} L\delta R$. On the other hand, calculating the derivative of F_{t} with respect to R, there is a term $\sim \sigma_{\mathrm{v}} L\, \delta R$. Therefore, the pressure work term is of order $R/R_{\mathrm{sphere}} \sim$ 20 nm/10 μm$\sim 2 \times 10^{-3}$ smaller than the tension-term in F_{t}, and we thus drop it. R is then found by minimizing F_{t}:

$$\left(\frac{\partial F_{\mathrm{t}}}{\partial R}\right)_{L,N_{\mathrm{l}},N_{\mathrm{p}}} = 0\,. \tag{S23}$$

Differentiating Eq. S7 leads to

$$\frac{1}{2R^2}\left(\kappa + 2\overline{\kappa}\,\phi_{\mathrm{t}}\right) - \frac{\overline{\kappa}\overline{C}_{\mathrm{p}}}{R}\,\phi_{\mathrm{t}} - \sigma_{\mathrm{t}} = 0\,. \tag{S24}$$

Eliminating σ_{t} using Eq. S19, an equation for R in terms of the vesicle tension σ_{v} and the tube and vesicle protein area fractions is found:

$$\frac{1}{2R^2}\left(\kappa + 2\overline{\kappa}\,\phi_{\mathrm{t}}\right) - \frac{\overline{\kappa}\overline{C}_{\mathrm{p}}}{R}\,\phi_{\mathrm{t}} - \sigma_{\mathrm{v}} - \frac{k_{\mathrm{B}}T}{a_{\mathrm{l}}}\left[\ln\left(\frac{1-\phi_{\mathrm{t}}}{1-\phi_{\mathrm{v}}}\right) + \left(1 - \frac{a_{\mathrm{l}}}{a_{\mathrm{p}}}\right)(\phi_{\mathrm{t}} - \phi_{\mathrm{v}})\right] = 0\,. \tag{S25}$$

To determine R as a function of vesicle tension, σ_{v}, for given ϕ_{v}, one must simultaneously solve the coupled equations for $\phi_{\mathrm{t}} = S\phi_v$ and R, given by Eqs. S20 and S25.

An approximate, analytical expression for R as a function of σ_{v} that gives qualitative agreement with experiments for ϕ_{v} between 2 to 5% can be obtained by taking, for simplicity, $\gamma = 1$. Also, we assume $\phi_{\mathrm{v}} \ll 1$, and $\overline{\kappa} \gg \kappa$ in Eq. S25; these conditions are verified experimentally. Under these assumptions and for non-zero σ_{v}, it can be first shown from Eq. S20 that $\phi_{\mathrm{t}} \simeq 1 - \frac{1}{\phi_{\mathrm{v}}}\exp\left[-\frac{\overline{\kappa}a_{\mathrm{p}}}{k_{\mathrm{B}}T}C\left(\overline{C}_{\mathrm{p}} - C\right)\right]$. The tube tension is then given by $\sigma_{\mathrm{t}} \simeq \sigma_{\mathrm{v}} - \frac{k_{\mathrm{B}}T}{a_{\mathrm{p}}}\ln\phi_{\mathrm{v}} - \overline{\kappa}C\left(\overline{C}_{\mathrm{p}} - C/2\right)$. Inserting these expressions into Eq. S24, one finds

$$R \simeq \sqrt{\frac{\kappa + \overline{\kappa}}{2\left(\sigma_{\mathrm{v}} - \frac{k_{\mathrm{B}}T}{a_{\mathrm{p}}}\ln\phi_{\mathrm{v}}\right)}}\,. \tag{S26}$$

From this expression we find that $\partial R/\partial\phi_{\mathrm{v}} > 0$, explaining why experimentally we see that, for moderate to high σ_{v}, the radius increases with increasing ϕ_{v} (see Fig. 3B). Second, it also confirms why the tube radius, in the presence of protein, is more weakly dependent on σ_{v} than a bare membrane: taking $\phi_{\mathrm{v}} = 0.03$, $k_{\mathrm{B}}T/a_{\mathrm{p}} = 0.08$ mN m^{-1}, we see that $-\frac{k_{\mathrm{B}}T}{a_{\mathrm{p}}}\ln\phi_{\mathrm{v}} \approx 0.3$ mN m^{-1}, and thus this term dominates the denominator above for $\sigma_{\mathrm{v}} < 0.1$ mN m^{-1}.

4.2 Pulling force

To determine the force, f, acting along any cross-section of the tube, we equate the work done in changing the length, L, of a cylindrical portion of tube of fixed number of lipids and proteins to the work done by f:

$$\left(\frac{\partial F_{\rm t}}{\partial L}\right)_{R,N_{\rm l},N_{\rm p}} \delta L = f\,\delta L\,. \qquad \text{S27}$$

Note that in the above operation R is held constant. Thus, while there is a change of volume associated with changing L and fixing R, we neglect the associated pressure work done for the reason mentioned earlier. The force is thus given by

$$f = \left(\frac{\partial F_{\rm t}}{\partial L}\right)_{R,N_{\rm l},N_{\rm p}}\,. \qquad \text{S28}$$

Differentiating the expression for the tube free energy, Eq. S7, with respect to L, after some algebra we find

$$f = 2\pi\,R\,\left(\frac{\kappa}{2R^2} + \sigma_{\rm t}\right)\,. \qquad \text{S29}$$

This expression for the force is the same as what one obtains for a bare membrane, except that $\sigma_{\rm t}$ appears instead of $\sigma_{\rm v}$. Note that an expression equivalent to Eq. S29 can be obtained by eliminating $\sigma_{\rm t}$ using Eq. S25:

$$f = 2\pi\left(\frac{\kappa + \overline{\kappa}\,\phi_{\rm t}}{R^2} - \frac{\overline{\kappa}\,\overline{C}_{\rm p}\,\phi_{\rm t}}{R}\right) \qquad \text{S30}$$

Therefore, to determine the force, f, as a function of $\sqrt{\sigma_{\rm v}}$—to compare it with the bare membrane expression $f = 2\pi\sqrt{2\kappa\sigma_{\rm v}}$—, R and $\phi_{\rm t}$ must be first found by solving Eqs. S20 and S25 numerically for given $\sigma_{\rm v}$.

An analytical expression for f can be obtained under the same approximations leading to Eq. S26. Using Eq. S26 and $\phi_{\rm t} \simeq 1$, we find

$$f \simeq 2\pi\sqrt{2\left(\kappa + \overline{\kappa}\right)\left(\sigma_{\rm v} - \frac{k_{\rm B}T}{a_{\rm p}}\ln\phi_{\rm v}\right)} - 2\pi\overline{\kappa}\,\overline{C}_{\rm p}\,. \qquad \text{S31}$$

The above expression yields $\partial f/\partial\phi_{\rm v} < 0$, confirming the experimental observation that, for given tension, the pulling force decreases with increasing $\phi_{\rm v}$; see Fig. 3A.

Interestingly, an estimate of the radius of spontaneously formed tubes (invaginations) can be made using Eqs. S26 and S31. Solving $f = 0$ first yields the tubulation tension

$$\sigma_{\rm v}^0 = \frac{\overline{\kappa}^2\,\overline{C}_{\rm p}^2}{2\left(\overline{\kappa} + \kappa\right)} + \frac{k_{\rm B}T}{a_{\rm p}}\ln\phi_{\rm v}\,. \qquad \text{S32}$$

Equation S32 resembles a result from recent work on spontaneous membrane tubulation of by bound endophilin [4]. The functional dependence of the critical tension on protein area fraction obtained in Ref. [4] is different than the above expression, though the trend ($\sigma_{\rm v}^0$ increases with increasing $\phi_{\rm v}$) is the same. The difference can be attributed to the experimental approach: in Ref. [4], perturbations of flat membranes by protein were studied, whereas here we are starting from a tubular state.

Finally, inserting Eq. S32 into Eq. S26 yields the spontaneous radius

$$R_0 = \left(\frac{\overline{\kappa} + \kappa}{\overline{\kappa}}\right) \overline{C}_{\rm p}^{-1} . \qquad \text{S33}$$

Thus, the radius of the protein-membrane "scaffold" is slightly greater than the intrinsic radius $\overline{C}_{\rm p}^{-1}$, due to the energetic cost of bending the underlying membrane.

4.3 Phase separation on the tube at low vesicle tension

In this section we show that at low tension and not too small $\phi_{\rm v}$ the tube radius is a multi-valued function of $\sigma_{\rm v}$, indicating bistable behaviour. To keep things tractable we assume $\gamma = 1$, though similar behavior is found numerically for any γ. In this case, an explicit form for $\phi_{\rm t}$ is obtained from Eq. S20. Inserting this expression into Eq. S25, writing $\sigma_{\rm v}$ as a function of C, and expanding to $O(C^2)$ and $O(\phi_{\rm v})$, we obtain

$$\sigma_{\rm v} \simeq \frac{1}{2} C^2 \left[\kappa - \phi_{\rm v}\,\overline{\kappa}\left(\frac{\overline{\kappa}\,\overline{C}_{\rm p}^2}{k_{\rm B}T/a_{\rm p}} - 1\right)\right] . \qquad \text{S34}$$

For $\phi_{\rm v} \to 0$, the tube curvature, $C \simeq \sqrt{\frac{2\sigma_{\rm v}}{\kappa - \phi_{\rm v}\,\overline{\kappa}\left(\frac{\overline{\kappa}\,\overline{C}_{\rm p}^2}{k_{\rm B}T/a_{\rm p}} - 1\right)}}$ [see Fig. 4D] tends to its bare value $\sqrt{2\sigma_{\rm v}/\kappa}$. However, taking $\kappa = 15\ k_{\rm B}T$, $\overline{\kappa} = 40\ k_{\rm B}T$, $\overline{C}_{\rm p} = 0.05\ {\rm nm}^{-1}$, and $a_{\rm p} = 50\ {\rm nm}^2$, the quantity in brackets becomes negative for $\phi_{\rm v} = \phi_{\rm v}^* \approx 6\%$, indicating that the tube curvature at zero tension changes discontinuously to a non-zero value at $\phi_{\rm v}^*$ (see Fig. 3B). Furthermore, for $\phi_{\rm v} \lesssim \phi_{\rm v}^*$, $\sigma_{\rm v}(C)$ is non-monotonic (with one local maximum and one local minimum) and there are two stable solutions for $R(\sigma_{\rm v})$, suggesting coexistence of cylindrical domains of different radii along the tube at a given tension. Below, we refer to the phase whose curvature vanishes (does not vanish) at zero $\sigma_{\rm v}$ as the "bare" (b) phase ("covered" (c) phase). The curvatures of the two phases are indicated $C_b = 1/R_b$ and $C_c = 1/R_c$. Similarly, the forces are f_b and f_c, and at equilibrium are given by Eq. S29, applied separately to the b and c phases.

Experimentally, one varies $\phi_{\rm v}$ and $\sigma_{\rm v}$ and we can determine, numerically, from our model the $b - c$ coexistence curve in the $(\phi_{\rm v}, \sigma_{\rm v})$-diagram. At equilibrium, Eq. S23 holds separately for the b and c phases. In addition, the axial force, f, is uniform along the tube. As a result, by numerically solving, for

given $\sigma_{\rm v}$, the three equations

$$\frac{\partial F_{{\rm t}b}}{\partial R_b} = 0 \qquad \text{S35a}$$

$$\frac{\partial F_{{\rm t}c}}{\partial R_c} = 0 \qquad \text{S35b}$$

$$f_b = f_c\,, \qquad \text{S35c}$$

the unknown quantities C_b, C_c, and the coexistence curve $\phi_{\rm v}(\sigma_{\rm v})$ can be found; see Fig. 4. In these equations, $F_{{\rm t}b}$ and $F_{{\rm t}c}$ refer to the tube free energy [Eq. S7] along the b and c phases. The protein densities $\phi_{{\rm t}b} = \phi_{\rm v}\, S_b$ and $\phi_{{\rm t}c} = \phi_{\rm v}\, S_c$ that figure in the free energy and force expressions are given by their equilibrium expression, Eq. S20.

In addition to the coexistence curve (Fig. 4D), the limits of metastability of the b and c phases can be determined from our model. Viewing $\sigma_{\rm v}$ as a function of C [See Eq. S25], the metastability limits are obtained by solving, for a particular tension $\sigma_{\rm v} = \sigma$, the values of $\phi_{\rm v}$ for which $\left.\frac{d\sigma_{\rm v}(C)}{dC}\right|_{\sigma_{\rm v}=\sigma} = 0$; See Fig. 4d. At the metastability limit of the b (c) phase, $\left.\frac{d^2\sigma_{\rm v}(C)}{dC^2}\right|_{\sigma_{\rm v}=\sigma} < 0 \ (> 0)$.

Finally, in the $(\phi_{\rm v},\, \sigma_{\rm v})$-diagram the coexistence and metastability limit curves converge at a critical point, obtained by solving $\frac{d\sigma_{\rm v}(C)}{dC} = 0$ and $\frac{d^2\sigma_{\rm v}(C)}{dC^2} = 0$. For our model, the critical point can be found exactly for the case $\gamma = 1$, yielding the critical point vesicle protein density

$$\phi_{\rm v}^{*} = \frac{\kappa}{2\overline{\kappa}} \exp\left(-\frac{\overline{\kappa}\,\overline{C}_{\rm p}^2\, a_{\rm p}/k_{\rm B}T - 3}{2} \right)\,. \qquad \text{S36}$$

The strong dependence of $\phi_{\rm v}^{*}$ on intrinsic curvature suggests a reason why weak protein curvature is needed to observe phase separation: for Amphiphysyin 1, $\overline{C}_{\rm p} \approx 1/3\ {\rm nm}^{-1}$ and the exponential factor above is vanishingly small, and it is therefore experimentally impossible to access low enough densities to observe phase separation.

It is important to note that our model predicts protein phase separation along the tube, even in the absence of direct protein-protein interactions. It is the nonlinear coupling between the protein density, $\phi_{\rm t}$, and the tube curvature, $1/R$, in the free energy, $F_{\rm t}$, that produces effective interactions leading to protein aggregation. The effect of direct interactions on phase separation can, nonetheless, be explored by restoring the interaction term in $f_{\rm m}$. Allowing for attractive interactions, $\chi < 0$, we find that their effect is to shift the coexistence and spinodal lines in the $(\phi_{\rm v},\, \sigma_{\rm v})$-diagram; see Fig. S9. As expected, such interactions favor the c phase at lower values of $\phi_{\rm v}$ than in their absence.

Supplementary references

[1] Sorre, B. *et al.* Nature of curvature coupling of amphiphysin with membranes depends on its bound density. *Proc Natl Acad Sci U S A* **109**, 173–8 (2012).

[2] Kwok, R. & Evans, E. Thermoelasticity of large lecithin bilayer vesicles. *Biophys J* **35**, 637–52 (1981).

[3] Landau, L. D. & Lifshitz, I. L. *Statistical Physics* (Butterworth Heinemann, London, 1980), 3rd edn.

[4] Shi, Z. & Baumgart, T. Membrane tension and peripheral protein density mediate membrane shape transitions. *Nat Commun* **6**, 5974 (2015).
